# From radiolabeling to receptor quantification: preclinical assessment of [^99m^Tc]Tc-carvedilol as a cardiac β-adrenoceptor probe

**DOI:** 10.3389/fphar.2025.1581598

**Published:** 2025-07-08

**Authors:** Hala F. Azhari

**Affiliations:** College of Medicine and Pharmacy, Umm Al-Qura University, Makkah, Saudi Arabia

**Keywords:** carvedilol, adrenergic receptors, technetium-99m, single-photon emission computed tomography nuclear imaging, cardiovascular disease, prognostic marker

## Abstract

**Introduction:**

Accurate cardiac adrenoceptor assessment is crucial for managing cardiovascular diseases. This study introduces a novel radiotracer, technetium-99m-labeled carvedilol ([^99m^Tc]Tc-carvedilol), which advances non-invasive cardiac receptor evaluation by improving traceability and myocardial tissue selectivity. Aimed at strengthening diagnostic precision, it optimizes a selective radioligand for quantifying cardiac adrenergic receptor sites.

**Methods:**

[^99m^Tc]Tc-carvedilol was synthesized *via* direct radiolabeling with technetium-99m, key parameters were optimized to maximize radiolabeling efficiency and ensure a reliable and reproducible [^99m^Tc]Tc-carvedilol complex. Biodistribution was rigorously evaluated *in vitro* and *in vivo*, emphasizing cardiac uptake, receptor occupancy, biodistribution, and clearance kinetics. Comparative analysis with [^131^I]iodocarvedilol and ^99m^Tc-sestamibi provided insights into advancements in detection efficiency and translational potential.

**Results:**

[^99m^Tc]Tc-carvedilol showed a radiolabeling efficiency of 96.5% ± 2.87%, with serum stability >92% at 24 h. Biodistribution studies in Swiss Albino mice (24 mice, aged 10–12 weeks, weighing 25 ± 3 g) revealed peak cardiac uptake (27.533% ± 0.931% injected dose per Gram of tissue (ID/g) within 15 min post-injection, alongside efficient blood clearance and minimal non-target tissue uptake (5.972% ± 0.131% ID/g organ) by 120 min. Docking analysis confirmed robust β_1_-adrenoceptors (−9.2 kcal/mol) *via* hydrogen bonds and hydrophobic and electrostatic interactions. Compared to [^131^I]iodocarvedilol and ^99m^Tc-sestamibi [^99m^Tc]Tc-carvedilol exhibited superior stability, targeting accuracy, and pharmacokinetics.

**Discussion:**

The enhanced selective cardiac uptake and favorable pharmacokinetics of [^99m^Tc]Tc-carvedilol position it as a promising agent for non-invasive cardiac receptor mapping, with the potential to improve diagnostic accuracy and specificity. Further clinical validation is essential to confirm its efficacy in detecting and evaluating cardiac pathologies.

## Graphical Abstract

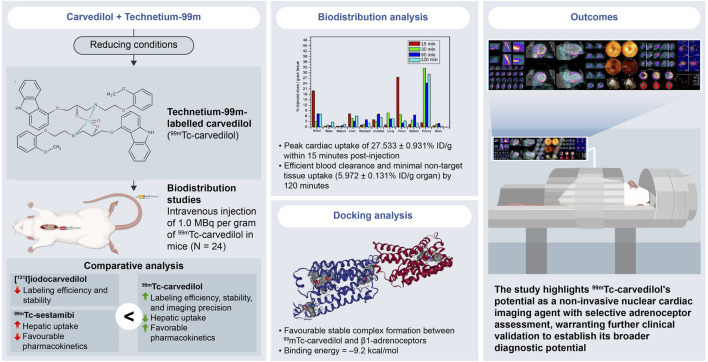

## 1 Introduction

Post-myocardial infarction (MI) stroke remains a severe and clinically significant complication, with mortality and morbidity rates exceeding those of strokes arising from non-cardiac etiologies (Sposato et al., 2020). Notably, strokes occur in approximately 1%–3% of patients within the first 3 months following MI (Putaala and Nieminen, 2018), a period marked by heightened vulnerability due to systemic pathophysiological shifts. Cardiac disease contributes nearly 20% of all ischemic stroke cases (Tsao et al., 2022), underscoring its pivotal role in cerebrovascular risk. This early post-MI phase is characterized by a hypercoagulable and pro-inflammatory milieu (Palasubramaniam et al., 2019), driven by myocardial necrosis, systemic cytokine release, and heightened sympathetic activity (Mourouzis et al., 2020). These mechanisms collectively enhance the propensity for thromboembolic cerebral events (Chen et al., 2017).

Adrenergic receptors (Bristow, 2000)—particularly β_1_ and β_2_ subtypes—are central to cardiovascular regulation, mediating heart rate, myocardial contractility, vascular tone, and peripheral resistance. β_1_ receptors (Bristow, 2000), predominantly located in cardiac tissue, increase heart rate and contractile force, potentially exacerbating myocardial oxygen demand and perfusion deficits during acute ischemic events such as MI (Gardner et al., 2016). β_2_ receptors (Bristow, 2000), by contrast, promote vasodilation and influence systemic vascular resistance, while α_1_ adrenergic receptors (Bristow, 2000), primarily found in vascular smooth muscle, induce vasoconstriction, raising blood pressure and afterload. Chronic stimulation of these receptors contributes to endothelial dysfunction, atherosclerotic progression, and sustained hypertension, pathophysiological changes that further elevate the risk of post-MI complications, including ischemic stroke (Ajoolabady et al., 2024). In ischemic and post-ischemic states, the expression and activity of adrenergic receptor subtypes (Strosberg, 1993) become dysregulated, reflecting the severity and progression of cardiovascular disease. Their altered signalling not only promotes hemodynamic instability and sympathetic overdrive but also engages pro-inflammatory and pro-thrombotic cascades (Zhang et al., 2021). Therefore, accurate mapping and quantification of cardiac adrenergic receptors offer transformative insights into myocardial remodelling, disease prognosis, and stroke susceptibility following MI. Such molecular characterization underpins the development of precision and personalized diagnostic strategies in cardiovascular medicine.

In this context, nuclear medicine and radiopharmaceuticals play an essential role in the diagnosis of diseases, infections, and malignancies that are otherwise challenging to detect using conventional imaging modalities. Nuclear medicine leverages targeted radiotracers to visualize physiological and molecular processes at the cellular level (Naqvi and Drlica, 2017), enabling early detection and characterization of pathological changes before anatomical abnormalities become apparent (Naqvi et al., 2010). Particularly in cardiology, radiopharmaceuticals have revolutionized diagnostic accuracy, risk stratification, and therapeutic monitoring, offering functional insights that complement morphological imaging (Ume and Erdoğan, 2012). Advances in radiopharmaceutical science have led to the development of highly specific agents capable of targeting distinct biological pathways, thereby enhancing the sensitivity and specificity of diagnostic imaging (Usmani et al., 2019).

Non-invasive imaging techniques (Dobrucki and Sinusas, 2010), including single-photon emission computed tomography (SPECT) and positron emission tomography (PET), offer valuable tools for visualizing adrenergic receptors and assessing cardiac function. However, the clinical utility of current radiopharmaceuticals (Cuocolo et al., 2011) is hindered by limited receptor specificity, metabolic instability, and high non-specific uptake in non-target tissues—factors that collectively compromise imaging precision and diagnostic reliability. This limitation is particularly consequential in the post-MI setting (Zhao et al., 2020), where precise evaluation of myocardial adrenergic receptor activity is critical for early risk stratification and guiding diagnostic interventions. Despite the availability of tracers (Manabe et al., 2018) such as ^123^I-MIBG and ^11^C-hydroxyephedrine, which are primarily designed to assess sympathetic nervous system activity with left ventricular dysfunction, there remains a significant gap in the development of agents that selectively target myocardial β_1_-adrenoceptors—key mediators of cardiac function and central contributors to post-MI remodeling. While tracers like ^11^C-CGP12177 have been introduced (Manabe et al., 2018), their limited clinical translation and lack of robust data on receptor-specific imaging in ischemic myocardium underscore the unmet need for pharmacologically selective, metabolically stable agents capable of mapping β_1_-adrenoceptor distribution with high fidelity.

Beta-blockers, particularly carvedilol (Dulin and Abraham, 2004), a non-selective beta-blocker with additional alpha-1 antagonistic and antioxidant properties, exhibit a unique pharmacological profile that lends itself to cardioprotective functions. Carvedilol’s high affinity for both β_1_ and β_2_ receptors, combined with moderate alpha-1 blockade, contributes to its ability to reduce oxidative stress, prevent mitochondrial dysfunction, and attenuate cardiomyocyte apoptosis under ischemic conditions. These properties have made it a mainstay in the management of chronic heart failure and post-MI care (Dulin and Abraham, 2004). Yet, despite its broad clinical utility, the potential of carvedilol as a radiolabeled imaging agent remains underexplored.

Radiographic quantification of β-adrenergic receptors *via* myocardial perfusion imaging (Boschi et al., 2022) is a sophisticated approach for refining diagnostic interventions. Radiometric quantification of β_1_ receptors is promising as an independent prognostic marker for cardiovascular disorders. Although an array of radioligands have been developed for cardiac imaging using SPECT and PET (Cuocolo et al., 2011), there remains a need for radioligands with high affinity, specificity, and metabolic stability to improve diagnostic accuracy. These attributes render [^99m^Tc]Tc-carvedilol exceptionally suited for precise and timely diagnostic assessments in clinical settings. When radiolabeled with technetium-99m, carvedilol not only retains its high affinity for myocardial β-adrenergic receptors but also benefits from the ideal nuclear properties of ^99m^Tc—including its optimal gamma emission energy (140 keV), short half-life (∼6 h), and broad clinical availability (Rathmann et al., 2019). These characteristics facilitate high-resolution imaging with low radiation exposure, enabling receptor-specific cardiac evaluation and enhancing its potential as a robust radiodiagnostic agent.

This study aimed to conduct a comprehensive preclinical assessment of [^99m^Tc]Tc-carvedilol in cardiac applications by (1) developing and optimizing a technetium-99m-labeled carvedilol compound, and (2) evaluating its concentration, biodistribution, and receptor binding affinity to quantify β-adrenoceptor density in heart tissue; receptor occupancy experiments were designed to confirm tracer specificity and its correlation with regional β-adrenoceptor density through competitive displacement by unlabeled carvedilol. It was hypothesized that [^99m^Tc]Tc-carvedilol, due to its high affinity for cardiac β-adrenoceptors and the favorable imaging properties of technetium-99m, would exhibit selective myocardial accumulation, enabling accurate quantification of receptor distribution.

## 2 Methods

### 2.1 Ethical approval

The *in vitro* and *in vivo* study protocols were conducted in full compliance with the relevant regulations and guidelines established by the Egyptian Atomic Energy Authority (Cairo, Egypt) (IAEA, 2021). Furthermore, all experimental procedures received ethical approval from the Biomedical Research Ethics Committee at Umm Al-Qura University (Approval No. HAPO-02-K-012-2025-05-2707) and the Animal Ethics Committee of the Egyptian Atomic Energy Authority (Approval No. EAEA/2022/188).

### 2.2 Chemicals and reagents

Carvedilol (molecular weight, 406.474 g/mol (2RS)-1-[9H-carbazol-4-yloxy]-3-[2-[2-ethoxypheoxy]amino]propan-2-ol) (Beattie et al., 2013) was sourced from the Memphis Pharmaceutical Company, Egypt, and all other chemicals (analytical reagents) were procured from Merck, Egypt. Technetium-99m was eluted as ^99m^TcO_4_^−^ from a 99Mo/^99m^Tc generator supplied by Gentech, Turkey. Unless otherwise stated, all chemicals were used directly without further purification, and deionized water was used in all experiments for solution preparation, dilution, and washing purposes. Radioactivity measurements were conducted using a well-type NaI(Tl) (Al Oraini, 2018) crystal coupled to an SR-7 scaler rate-meter provided by the Ludlum Measurements Inc., Texas, United States. Whatman No. 1 paper chromatography (Whatman International Ltd., Maidstone, Kent, United Kingdom) was used for the analysis.

### 2.3 Labeling of carvedilol

The [^99m^Tc]Tc-carvedilol complex was synthesized through the direct reaction of carvedilol with technetium-99m under reducing conditions facilitated by stannous chloride (SnCl_2_·2H_2_O). Specifically, 1.0 mL of ^99m^Tc eluate, containing 195 MBq of ^99m^TcO_4_^−^ was added to the above mixture and allowed to react at 25 °C for up to 24 h to produce the [^99m^Tc]Tc-carvedilol complex. The efficiency of radiolabeling purity and stability was optimized by examining various reaction parameters affecting the [^99m^Tc]Tc-carvedilol radiochemical yield under the following conditions: (1) concentration effect of carvedilol; (2) amount of SnCl_2_·2H_2_O reducing agent content; (3) reaction time; (4) pH of the reaction medium; and (5) concentration and biodistribution studies.

### 2.4 Determination of radiochemical purity

The radiochemical purity (RCP) of the radiolabeled complex [^99m^Tc]Tc-carvedilol, was evaluated using a paper chromatography technique (Kumari et al., 2022) on Whatman No. 1 filter strips. For each analysis, two chromatography strips (1.0 cm in width and 13 cm in length) were prepared. A 1.0 to 2.0 µL sample of the reaction mixture was carefully applied 2.0 cm from the lower edge of each strip, allowing the spot to dry completely before further processing.

Each strip was developed in a different solvent system to differentiate the chemical forms of technetium and assess the RCP of the labeled compound. One strip was developed using acetone, which effectively facilitates the migration of free pertechnetate (^99m^TcO_4_^−^) to the solvent front due to its polar and non-complexing nature. This allows for accurate quantification of unbound ^99m^Tc species, an essential parameter in radiopharmaceutical quality control. The second strip was developed using a solvent mixture of ethanol:water:hydroxide in a 2:5:1 ratio, specifically chosen to retain the reduced hydrolyzed technetium (^99m^Tc-colloids) at the origin while allowing migration of the radiolabeled complex. These solvent systems were not arbitrarily selected but rather optimized based on their established efficacy in technetium-99m radiopharmaceutical analyses (Martini et al., 2016).

The ethanol:water:hydroxide system provides an alkaline medium that stabilizes reduced hydrolyzed technetium in its colloidal form, facilitating clear separation from the labeled species and free pertechnetate. The hydroxide component was freshly prepared by diluting a concentrated sodium hydroxide solution to a working molarity of approximately 0.1 M, immediately before use, to ensure consistent solvent performance and minimize degradation or carbonate formation, which can alter chromatographic profiles. This dual-solvent chromatographic strategy enabled clear identification of radioactivity peaks corresponding to free pertechnetate at the solvent front in acetone, hydrolyzed-reduced technetium remaining at the origin in ethanol:water:hydroxide, and the desired [^99m^Tc]Tc-carvedilol complex migrating to distinct Rf positions. By calculating the proportion of total radioactivity associated with the desired radiolabeled complex *versus* impurities, the RCP was accurately determined. This methodological rigor is crucial for validating the integrity and stability of the [^99m^Tc]Tc-carvedilol formulation before proceeding with biological evaluation.

The RCP was further assessed by high-performance liquid chromatography (HPLC) (Abdu Hussen, 2022) using a Hitachi model (Japan). The HPLC procedure involved injecting 10 µL of the purified [^99m^Tc]Tc-carvedilol complex into an Alphabond RP-C18 column (300 × 3.9 mm) and detecting it using an ultraviolet spectrophotometer (SPD-6A) (Singhal et al., 2024) set at 282 nm. This setup measured the absorbance of pure carvedilol (control) and the [^99m^Tc]Tc-carvedilol complex (experimental) at their characteristic wavelengths to verify conjugation. Successful binding was indicated by an altered absorbance profile of the complex compared to that of free carvedilol, suggesting a change in the electronic environment due to ^99m^Tc binding. The mobile phase, a 50:50 v/v mixture of acetonitrile and 0.3 M potassium dihydrogen phosphate (pH 3.2), was delivered at a flow rate of 1.2 mL/min.

Mass spectrometry analysis (Zhang et al., 2023) was performed by measuring the mass (m) to charge (z) ratio (m/z) to confirm the molecular composition by identifying the characteristic (m/z) ratio of [^99m^Tc]Tc-carvedilol (experimental complex). For carvedilol (control), in the absence of technetium, the m/z value typically appears at approximately 406.474 g/mol (carvedilol molecular weight). After conjugation with ^99m^Tc, mass spectrometry displays a unique m/z peak corresponding to the complex, often with an increase reflecting the mass of the ^99m^Tc component. By obtaining the spectra of both pure carvedilol and the [^99m^Tc]Tc-carvedilol complex, any shift in the m/z values confirms the presence of technetium and successful conjugation, validating the suitability of the radiolabeled product for subsequent research applications.

### 2.5 Stability in serum

To preliminarily assess the *in vivo* stability of the [^99m^Tc]Tc-carvedilol complex, an *in vitro* stability study was conducted using commercially sourced human serum obtained from certified vendors (Sigma-Aldrich, Thermo Fisher Scientific). These suppliers adhere to stringent ethical standards and guidelines, ensuring serum is collected from screened, healthy donors in compliance with Institutional Review Board and Institutional Animal Care (National Institute of Health, 2024). Human serum was selected as a biologically relevant matrix that closely simulates the extracellular environment *in vivo*, particularly the circulatory system, where radiopharmaceuticals initially interact with plasma proteins, enzymes, and other biomolecules. This model is widely accepted in the early-stage evaluation of novel radiolabeled compounds, offering predictive insight into the compound’s stability, metabolic degradation, and resistance to transchelation prior to *in vivo* application.

The *in vitro* stability assessment involved incubating a mixture of 1.0 mL of normal human serum and 0.5 mL of [^99m^Tc]Tc-carvedilol at 37 °C for up to 24 h. Aliquots (0.2 mL) were withdrawn at predefined intervals and analyzed using a paper chromatographic technique (Rathmann et al., 2019). Chromatographic separation was carried out using a Shimadzu system (Shimadzu Corporation) (Genzo Shimadzu Sr., Kyoto, Japan), comprising LC-9A pumps, a Rheodyne injector (Model 7125), and an ultraviolet spectrophotometer detector (SPD-6A, Shimadzu). The mobile phase consisted of acetonitrile and water (65:35) containing 0.1% trifluoroacetic acid, delivered at a flow rate of 1.0 mL/min. The rationale for selecting this specific mobile phase composition lies in its optimized polarity under reversed-phase chromatographic conditions, particularly suited for the lipophilic nature of carvedilol and its technetium-labeled derivative. The use of acetonitrile at 65% enhances the elution strength, enabling efficient and rapid separation of the compound with minimal retention time. The aqueous component ensures adequate polarity balance, facilitating interaction with the stationary phase. The addition of 0.1% trifluoroacetic acid plays a critical role in peak sharpening and reducing tailing by maintaining a low pH environment, which suppresses ionization of both analytes and potential interfering matrix components. This acidic condition promotes consistent retention behavior, improved signal resolution, and reproducibility. Such a combination has been widely applied in analytical separations of β-blockers and radiopharmaceuticals, where RCP and stability are essential parameters (Winkler et al., 1985). Separated fractions were collected for up to 12 min, and the radiochemical integrity was subsequently evaluated using a well-type NaI(Tl) (Al Oraini, 2018) detector linked to a single-channel analyzer.

### 2.6 Concentration, biodistribution, and receptor occupancy studies

For biodistribution and receptor occupancy studies, 24 healthy adult Swiss albino mice (12 males and 12 females, aged 10–12 weeks, weighing 25–30 g) were obtained from the Egyptian National Research Center. Mice outside the 25–30 g weight range were excluded to ensure consistency in the biodistribution outcomes. Prior to the study, animals were housed in groups of four with *ad libitum* access to food and water and underwent a 2-week acclimatization period. After this adaptation phase, the mice were randomly assigned to the study groups using the lot-drawing method.

Each mouse received a standardized intravenous injection of 1.0 MBq per Gram of [^99m^Tc]Tc-carvedilol *via* the tail vein and was housed individually. Given ^99m^Tc’s physical half-life of approximately 6 h, shorter imaging and sampling intervals are generally preferred to maximize radioactive signal fidelity and minimize decay-related variability. At 24 h post-injection, the residual ^99m^Tc activity could fall below quantifiable thresholds, potentially compromising the precision of biodistribution data. To prevent this, all data collection was restricted to early post-injection intervals—specifically 0.5, 2, 4, and 6 h—when radioactivity levels remain within optimal detection ranges for gamma counting. By omitting the 24 h time point altogether, the study design ensured that the radiotracer signal would be robust enough to yield statistically reliable biodistribution profiles without being confounded by the isotope’s exponential decay. Additionally, prior pilot studies were conducted to empirically determine the time window during which the radiochemical integrity and quantification signal-to-noise ratio remained analytically valid. These informed the exclusion of late-phase sampling and reinforced confidence in the reliability of kinetic data collected within the defined 6 h observation window. Therefore, six mice were euthanized at each selected time point *via* standardized cervical dislocation performed by trained personnel in accordance with institutional animal care guidelines. The procedure followed the AVMA Guidelines for the Euthanasia of Animals (Kirkwood, 2013), ensuring it was rapid, humane, and reproducible. To ensure procedural consistency, all cervical dislocations were performed by the same experienced researcher, following pre-established protocols that involved correct animal positioning, anatomical landmark identification, and verification of death through cessation of heartbeat and respiration. Immediately postmortem, body weight was recorded, and blood was collected *via* cardiac puncture.

Stringent precautions were implemented throughout the preparation and handling of [^99m^Tc]Tc-carvedilol to ensure the maintenance of its RCP and to minimize decay-related data distortion. Organ harvesting included the collection of the heart, liver, lungs, kidneys, spleen, stomach, intestines, muscle (right leg), bone, and brain. Following dissection, tissues were gently rinsed with isotonic saline to remove residual blood, promptly weighed, kept at low temperatures, and immediately subjected to gamma counting to preserve radiochemical integrity and avoid post-collection decay artifacts. No storage or freezing was used to prevent degradation or leakage of [^99m^Tc]Tc-carvedilol. Sample processing occurred in a shielded, on-site laboratory adjacent to the dissection area, ensuring minimal delay—typically less than 2 to 3 minutes—between collection and measurement.

The RCP of [^99m^Tc]Tc-carvedilol was confirmed both pre- and post-harvesting using thin-layer chromatography and gamma spectroscopy to ensure that technetium remained stably chelated. This dual-stage verification eliminated concerns over radiolytic degradation or detachment from the carrier molecule. The thin-layer chromatography results consistently demonstrated a single radiochemical species corresponding to intact [^99m^Tc]Tc-carvedilol, with RCP exceeding 95% in all collected samples (Mercado et al., 2023). Additionally, dose calibrators were employed to normalize gamma readings to the injection time, correcting for any residual decay and ensuring temporal consistency of biodistribution results. This confirmed that the radioactivity detected in organs reflected bound radiocomplex, rather than free pertechnetate or degraded species. This analytical approach is consistent with established protocols in radiopharmaceutical quality control (Maioli et al., 2017).

Strict adherence to validated protocols and internationally recognized quality control standards was maintained to preserve the radiochemical stability and functional integrity of the [^99m^Tc]Tc-carvedilol compound throughout its intended application. This rigorous quality assurance process was essential for ensuring the reliability, reproducibility, and accuracy of the study’s outcomes. To further enhance the validity of biodistribution results, time points were carefully selected based on the known pharmacokinetic profile of [^99m^Tc]Tc-carvedilol. Rapid post-collection analysis was performed to mitigate any potential degradation or radiolytic loss. All experimental procedures—including organ harvesting and radioactivity measurements—were carried out under blinded conditions to eliminate procedural bias and uphold the scientific integrity of the investigation.

### 2.7 Outcome measures

To optimize the binding and imaging quality of the [^99m^Tc]Tc-carvedilol compound, specific increments in conditions were systematically tested, allowing the identification of optimal conditions for purity, stability, and efficacy. The primary outcome was the successful labeling of carvedilol (control). Secondary outcome measures aimed to determine the optimal conditions for radioligand labeling yield efficiency, evaluated through the following steps: (1) varying the mass of carvedilol ligand (10 to 220 µg); (2) adjusting the amount of SnCl_2_.2H_2_O (10 to 200 µg) as a reducing agent; (3) testing different reaction times (0 to 120 min); (4) altering the pH of the reaction medium (3 to 10); (5) monitoring concentrations, conducting biodistribution, and receptor occupancy studies; and (6) performing statistical analysis to assess radiochemical yield (experimental complex) purity and stability. These factors were comprehensively assessed using *in vitro* human serum and *in vivo* Swiss Albino experimental model endpoints.

### 2.8 Statistical analysis

To ensure methodological rigor and reproducibility, both experimental and computational components of the study were supported by a structured statistical framework. A total of 24 healthy albino mice (12 males, 12 females), aged 10–12 weeks and weighing 25 ± 3 g, were selected based on uniform health status, age, and weight to minimize inter-animal variability. Animals were maintained under controlled laboratory conditions (12-h light/dark cycle, standardized humidity, and temperature) with unrestricted access to food and water. For biodistribution analysis, animals were randomly assigned to four experimental groups, each consisting of six mice, each representing a distinct post-injection time point (15, 30, 60, and 120 min). The rationale for selecting these specific time intervals was to comprehensively capture the pharmacokinetic and receptor-binding dynamics of the [^99m^Tc]Tc-carvedilol complex across the early distribution, peak uptake, and clearance phases. The 15- and 30-min intervals reflect the initial vascular and tissue perfusion phases, providing critical insight into the tracer’s rapid uptake in high-perfusion organs such as the heart and liver. The 60 min time point represents the anticipated peak receptor binding phase, where optimal myocardial accumulation is expected due to carvedilol’s high affinity for β_1_-adrenergic receptors in cardiac tissue. The 120 min time point allows evaluation of the tracer’s clearance kinetics, potential redistribution, and stability within peripheral tissues, thus enabling assessment of its biological half-life and specificity retention over time. This time-course approach ensures that kinetic modeling of tissue uptake and clearance can be achieved with adequate temporal resolution to inform future imaging protocol development. This grouping strategy was designed to detect meaningful differences in biodistribution parameters while achieving a statistical power of 80% (Serdar et al., 2021).

The rationale for selecting a statistical power of 80% lies in its widespread acceptance as a benchmark in biomedical research for balancing Type II error control with practical feasibility. A power of 80% ensures that there is an 80% probability of detecting a true effect, if one exists, thereby reducing the risk of false-negative results. This threshold is particularly critical in preclinical pharmacokinetic studies where effect sizes may vary across tissues and time points. Moreover, sample size calculations based on 80% power align with established norms in radiopharmaceutical biodistribution studies (Smith et al., 2009), optimizing both statistical sensitivity and ethical use of animal models. This stratification enabled temporal mapping of [^99m^Tc]Tc-carvedilol pharmacokinetics and receptor interaction across cardiac and peripheral tissues, providing high-resolution insights into tracer dynamics.

Radioactivity levels in excised organs were quantified using a well-type NaI(Tl) (Al Oraini, 2018) crystal coupled to an SR-7 scaler rate meter. The RCP of [^99m^Tc]Tc-carvedilol was assessed through chromatographic techniques, and the mean RCP values from four independent preparations were recorded and calculated using the following formula:

$$\%\text{ reduced hydrolyzed technetium}=100\;‐\;\left(\%\text{ colloid}+\%\text{ free pertechnetate}\right)$$

Biodistribution data were expressed as mean percent injected dose per Gram of tissue (%ID/g) ± standard deviation (SD). For evaluating lipophilicity, the partition coefficient (Duffield et al., 2021) (log *P*) was calculated using a two-phase octanol–water system based on the ratio of radioactivity measured in each layer and calculated using the following equation:

$$\log P=\log\left(\text{radioactivity}\text{in the organic layer}\right)/\left(\text{radioactivity in the aqueous layer}\right)$$

In competitive binding (receptor-blocking) assays, excess non-radioactive carvedilol was administered 30 min before tracer injection to assess β1-adrenoceptor specificity. Mean uptake values were compared between blocked and unblocked groups. Inferential statistical comparisons were conducted using a two-tailed Student’s t-test (Owen, 1965), and significance was determined at *P* ≤ 0.05.

### 2.9 Computational docking analysis

Geometry-optimized structures of [^99m^Tc]Tc-carvedilol were generated using Marvin Space and energy-minimized *via* ChemAxon 6.1.4 (Cheminformatics, Budapest, Hungary) (ChemAxon) Subsequent *in silico* analysis was performed using ChemBioOffice Ultra V14 software (Software.informer, 2014), which facilitated the visualization and refinement of conformationally stable ligand poses. The cardiac β1-adrenoceptor (PDB ID: 2W4O) was retrieved from the Research Collaboratory for Structural Bioinformatics Protein Data Bank (RCSB PDB) and was selected as the docking target, and iGemDock 2.1 software (Hsu et al., 2011) was employed to simulate ligand-receptor binding. Molecular docking was employed to elucidate the potential binding sites, energetically favorable conformations, and key non-covalent interaction patterns—particularly hydrogen bonding and hydrophobic interactions—between [^99m^Tc]Tc-carvedilol and its cardiac adrenergic receptor target.

### 2.10 Study reporting

The Checklist for Animal Research: Reporting of *In Vivo* Experiments (Percie du Sert et al., 2020) was followed in conducting and reporting of this study.

## 3 Results

The RCP of a radiopharmaceutical product reflects the proportion of radioactivity in the desired radiolabeled form (Molavipordanjani et al., 2019), which is essential for diagnostic accuracy. Ideally, the primary radioactivity should be in the bound form, whereas unbound fractions, including free pertechnetate (^99m^TcO_4_-) and colloidal technetium, should be minimized, as they reduce the RCP and may compromise diagnostic precision. These unbound forms occur when technetium-99m does not fully bind to carvedilol during radiolabeling.

Paper chromatography (Kumari et al., 2022) and HPLC (Abdu Hussen, 2022) were used to quantify these impurities. In paper chromatography using acetone as the developing solvent, free pertechnetate migrates to the solvent front (Rf = 1), whereas [^99m^Tc]Tc-carvedilol and colloidal technetium remain at the origin. The reduced hydrolyzed technetium identified using an ethanol:water:hydroxide (2:5:1) solvent, also remained at the origin (Rf = 0), whereas other impurities moved with the solvent front. This separation distinguishes bound technetium from impurities, allowing for the precise calculation of the RCP.

**FIGURE 1:**
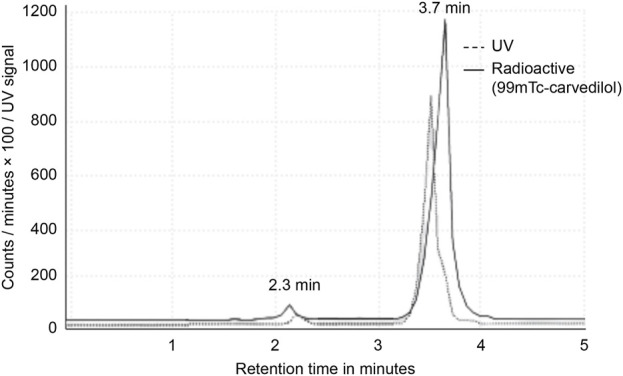
High-performance liquid chromatogram analysis of [^99m^Tc]Tc-carvedilol. Chromatograms of pure carvedilol (control) and the [^99m^Tc]Tc-carvedilol complex (experimental), both measured at 280 nm, were compared to visually confirm conjugation and assess conjugate purity through retention time shifts and peak integration analysis observed at 2.3 and 3.7 min, respectively. No significant peaks corresponding to free technetium or technetium oxide were detected, suggesting efficient conjugation and minimal free pertechnetate (^99m^TcO_4_^−^).

The RCP was then calculated by subtracting the percentage of free pertechnetate and colloidal technetium from the total radioactivity, yielding the fraction of technetium-99m successfully bound to carvedilol. HPLC analysis further confirmed the RCP, with free ^99m^TcO_4_- and [^99m^Tc]Tc-carvedilol showing distinct retention times at 2.3 and 3.7 min, respectively. The HPLC system’s effective separation of [^99m^Tc]Tc-carvedilol demonstrated its suitability for purification and quality control, as shown in Figure 1.

### 3.1 Effect of carvedilol amount for a selective radioligand

**FIGURE 2:**
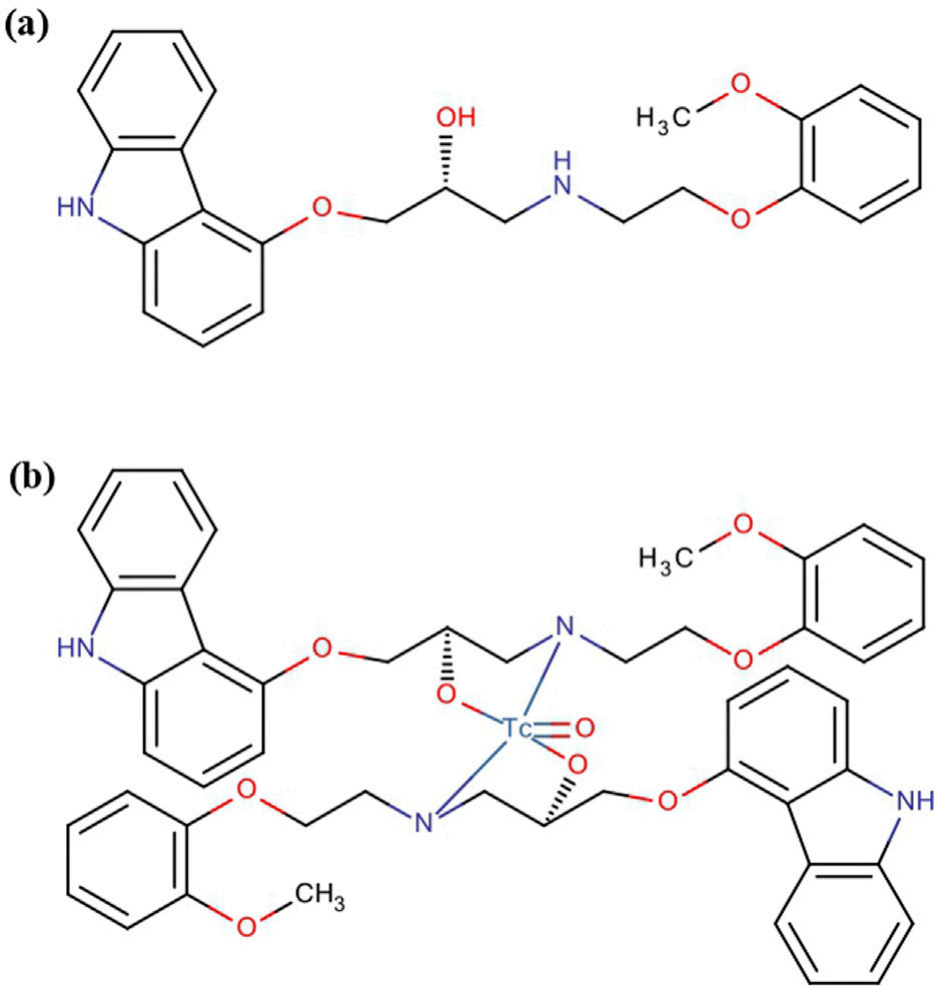
Pharmacochemical structure of **(a)** carvedilol drug and **(b)** the [^99m^Tc]Tc-carvedilol structure.

**FIGURE 3:**
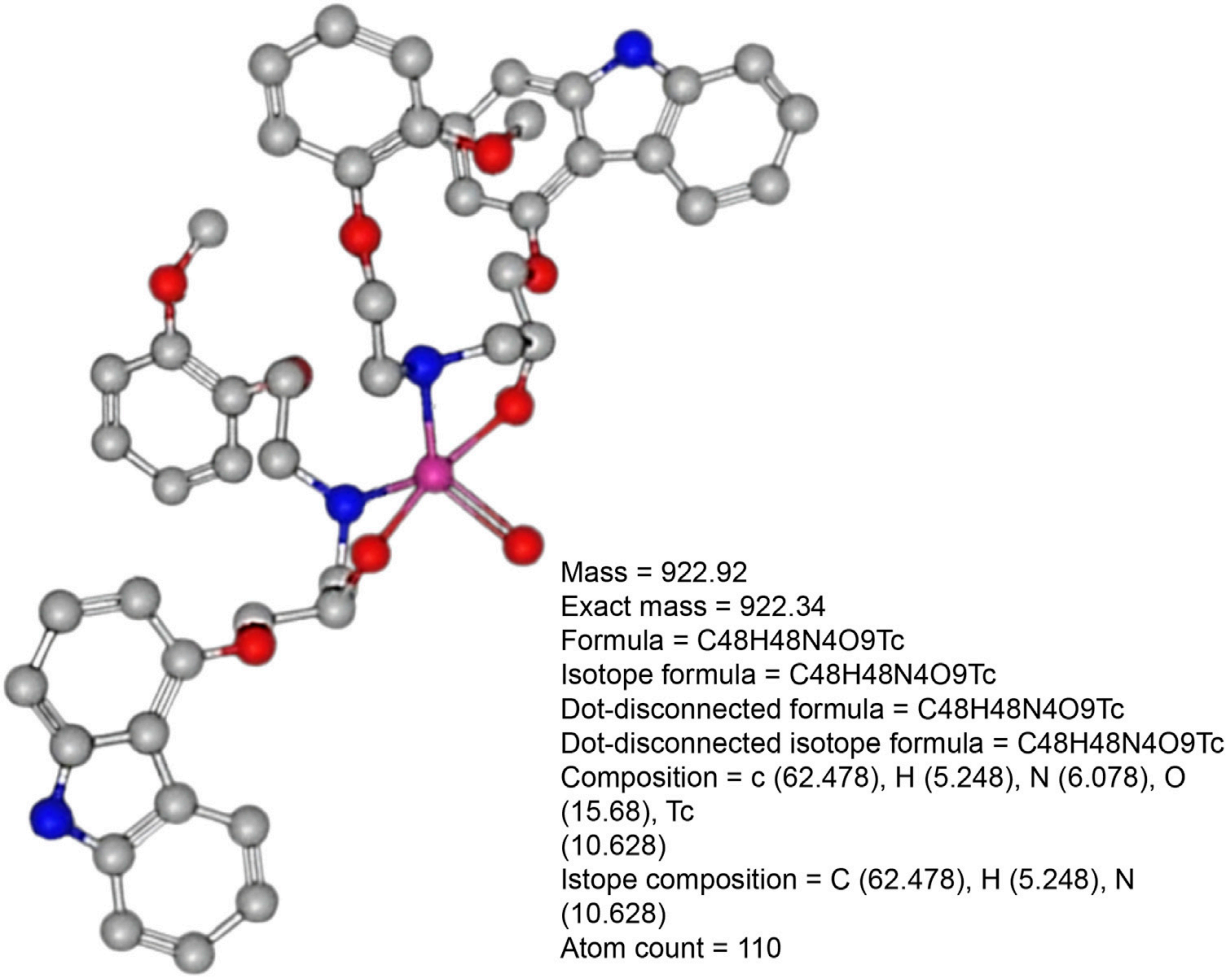
Optimized three-dimensional structure of [^99m^Tc]Tc-carvedilol electrical analysis.

Carvedilol was labeled with technetium-99m using a direct labeling technique, in which the reduced form of technetium-99 m reacted with carvedilol to form a labeled chelate. The influence of the electrical mass of carvedilol on the radiochemical yield and the optimized three-dimensional structure of [^99m^Tc]Tc-carvedilol are illustrated in Figure 2 and Figure 3., respectively.

**FIGURE 4:**
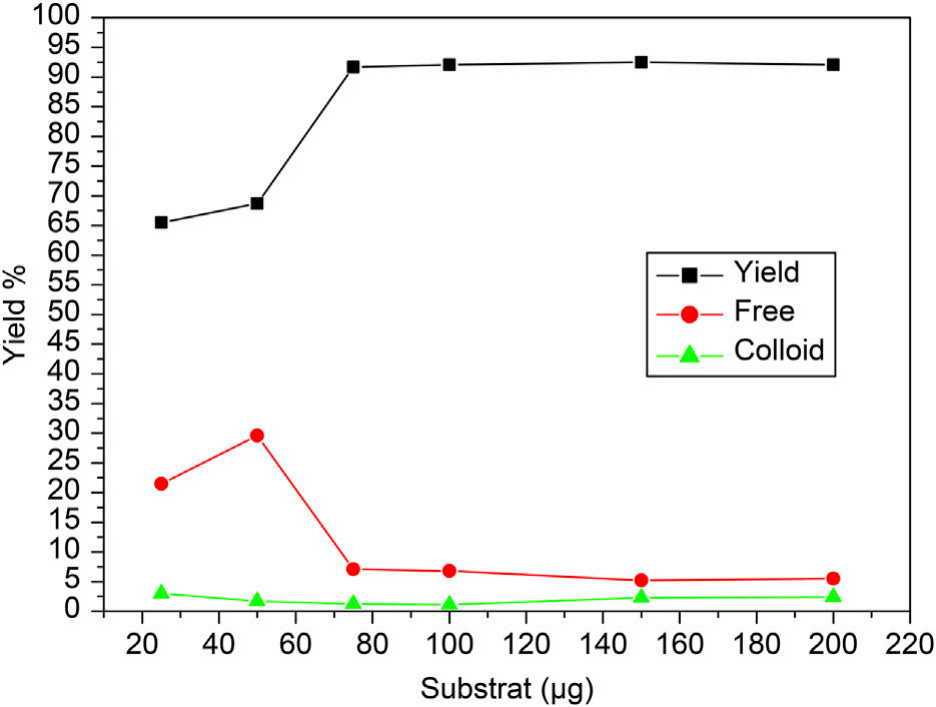
Radiochemical yield of the [^99m^Tc]Tc-carvedilol as a function of varying carvedilol concentrations. Line plots illustrate the influence of carvedilol concentration on the radiochemical yield of [^99m^Tc]Tc-carvedilol (black squares), unbound free pertechnetate (red circles), and colloidal impurities (green triangles). Radiolabeling was conducted under standardized conditions using varying amounts of carvedilol (x µg), 65 µg of SnCl_2_·2H_2_O as a reducing agent, and 1.0 mL (∼195 MBq) of [^99m^Tc]NaTcO_4_ at pH 7. Optimization of the ligand-to-metal molar ratio was critical to achieving high radiochemical purity and minimizing the presence of free ligand, which can interfere with chelation and lower labeling efficiency. Notably, increasing the ratio from 1:1 to 2:1 significantly enhanced labeling, achieving radiochemical purities up to 96%. Each data point represents the mean of four independent experiments conducted under identical conditions. A total of 24 healthy Swiss albino mice were employed (six per experiment) in subsequent biological evaluation. Error bars represent standard deviation (SD), indicating variability across replicates.

The reaction was conducted across a range of carvedilol mass (10 to 220 µg) to assess its effect on the radiochemical yield, free pertechnetate, and colloidal forms during the labeling of [^99m^Tc]Tc-carvedilol. The radiochemical yield of [^99m^Tc]Tc-carvedilol initially increased, reaching an optimal level at approximately 100 µg of the carvedilol drug ligand, beyond which the yield plateaued at approximately 96.5% ± 2.87%, ensuring complete complexation of reduced technetium-99m. Below this threshold, inadequate ligand amounts led to higher levels of reduced hydrolyzed technetium, reaching 65% at 50 µg of carvedilol. Conversely, employing >100 µg of the ligand did not improve the labeling yield, which remained stabilized around 96%. The percentage of free pertechnetate, which is inversely related to radiochemical yield, peaked at a lower carvedilol mass and diminished as the mass approached the optimal level. The formation of colloids, which remained relatively low and stable across the mass range, indicated that the formation of colloidal impurities was minimal under the tested conditions. This suggests that an optimized mass of carvedilol is critical for achieving maximum labeling efficiency with minimal radiochemical impurities, as illustrated in Figure 4.

### 3.2 Effect of SnCl_2_·2H_2_O reducing agent content

**FIGURE 5:**
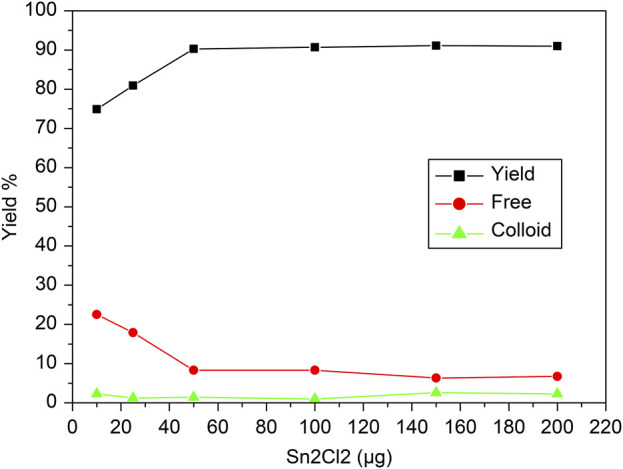
Radiochemical yield of the [^99m^Tc]Tc-carvedilol as a function of the reducing agent concentration (SnCl_2_·2H_2_O). Line plots illustrate the radiochemical yield of [^99m^Tc]Tc-carvedilol (black squares), unbound free pertechnetate (red circles), and colloidal impurities (green triangles) in response to varying concentrations of the reducing agent stannous chloride (SnCl_2_·2H_2_O). Radiolabeling was conducted under the following conditions: 100 µg carvedilol, variable SnCl_2_·2H_2_O (× µg), and 1.0 mL of [^99m^Tc]NaTcO_4_ (∼195 MBq) at pH 7.0. Each data point represents the mean yield from four independent experiments performed under identical experimental conditions. A total of 24 healthy Swiss albino mice (six per experiment) were subsequently used in biological validation studies utilizing these preparations. Error bars reflect the SD, indicating the extent of variation among replicates and ensuring analytical robustness.

The influence of the concentration of reducing agent, SnCl_2_·2H_2_O, on the labeling efficiency of [^99m^Tc]Tc-carvedilol is illustrated in Figure 5. The radiochemical yield was closely dependent on the concentration of SnCl_2_·2H_2_O in the reaction mixture. Initially, the labeling yield was 76% at 10 mmol of SnCl_2_·2H_2_O, indicating incomplete reduction of ^99m^TcO_4_^−^ and the presence of significant free pertechnetate (24%). However, as the mass of SnCl_2_·2H_2_O increased from 10 to 50 μg, the labeling yield increased significantly, reaching a peak efficiency of 92.5% ± 2.87%. Beyond this optimal mass, the yield declined because of the formation of tin colloids (8%), which competed with carvedilol for the reduced ^99m^Tc. This is evident from the stable, yet minimal levels of colloidal impurities observed across the range, confirming that the optimal amount of SnCl_2_·2H_2_O is crucial for maximizing RCP and minimizing undesirable species.

### 3.3 Effect of reaction time and stability

**FIGURE 6:**
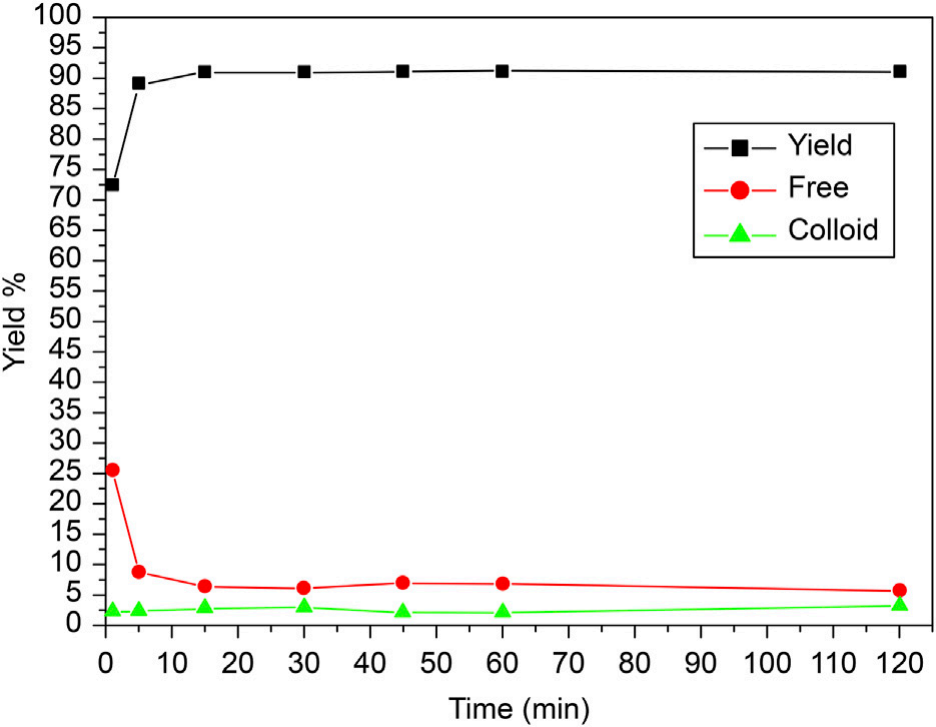
Radiochemical yield of the [^99m^Tc]Tc-carvedilol as a function of reaction time and stability. Line plots illustrate the radiochemical yield of [^99m^Tc]Tc-carvedilol (black squares), free pertechnetate (red circles), and colloidal impurities (green triangles) measured at varying reaction times. Radiolabeling was performed under standardized conditions: 100 µg carvedilol, 65 µg SnCl_2_·2H_2_O, and 1.0 mL of [^99m^Tc]NaTcO_4_ (∼195 MBq) at pH 7.0. Each data point represents the mean percentage yield obtained from four independent experiments conducted under identical conditions. A total of 24 healthy Swiss albino mice (six per experiment) were employed in downstream biodistribution studies using identically prepared radiolabeled formulations. The plotted trends depict the time-dependent evolution of radiolabeling efficiency and impurity formation. Error bars represent the SD, capturing the degree of experimental variability and reproducibility.

The reaction time for labeling carvedilol with ^99m^Tc played a crucial role in determining the efficiency of the labeling process. To assess the optimal usage window, the effect of time on the *in vitro* stability of [^99m^Tc]Tc-carvedilol complex was examined based on the reaction duration, as illustrated in Figure 6. Initially, at 0-min mark, the free technetium concentration was notably high. However, as the reaction progressed from 0 to 5 min, a significant decrease in free technetium was observed, indicating its effective reduction and incorporation into the [^99m^Tc]Tc-carvedilol complex. This trend suggests that sufficient time is required for the complete reduction of ^99m^TcO_4_^−^ and its subsequent binding to carvedilol. Concurrently, the yield of the labeled compound increased rapidly, achieving near-maximum efficiency within the first 5 min. Beyond this initial period, from 5 min onwards up to 120 min, the radiochemical yield stabilized, maintaining a high efficiency without fluctuation. The concentration of free technetium remained consistently low, indicating the sustained stability of the labeling process. Colloid formation was minimal throughout the reaction, suggesting that the reaction conditions effectively suppressed unwanted colloid production.

### 3.4 Effect of pH and purity

In this experiment, the pH was carefully adjusted to optimize the radiolabeling efficiency of the [^99m^Tc]Tc-carvedilol complex. The pH was controlled by adding buffer solutions or adjusting the reaction mixture with an acid or base as needed. Typically, solutions such as phosphate-buffered saline were used to maintain a stable pH, or small amounts of acidic (e.g., hydrochloric acid) or basic (e.g., sodium hydroxide) solutions were added incrementally.

**FIGURE 7:**
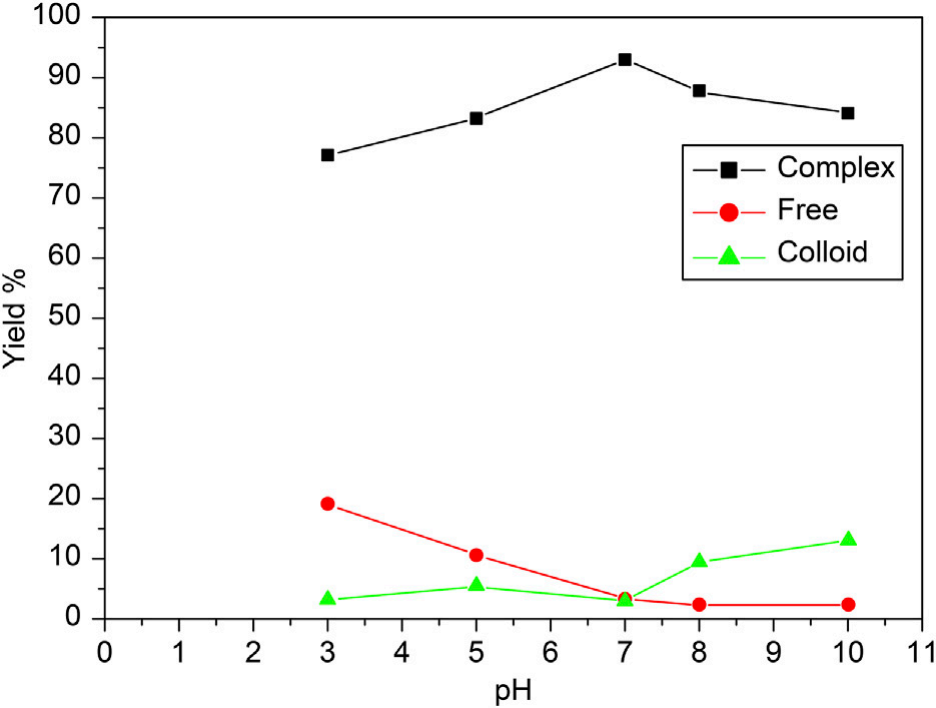
Radiochemical yield of the [^99m^Tc]Tc-carvedilol as a function of pH and purity. Line plots depict the percentage yields of [^99m^Tc]Tc-carvedilol (black squares), free pertechnetate (red circles), and colloidal species (green triangles) across varying pH conditions. Radiolabeling was performed using 100 µg carvedilol, 65 µg SnCl_2_·2H_2_O, and 1.0 mL of [^99m^Tc]NaTcO_4_ (∼195 MBq), with pH systematically adjusted. Each data point represents the mean percentage yield of four independent experiments, each validated through identical procedures. A total of 24 healthy Swiss albino mice (six per experiment) were employed to assess biodistribution parameters under the same radiochemical conditions. Error bars indicate the SD, reflecting variability in yield across replicates and highlighting the reproducibility of the labeling process. The observed trends demonstrate optimal radiochemical yield and minimal colloid or free pertechnetate formation near neutral pH.

The effect of the pH of the reaction mixture on the radiochemical yield, free technetium, and colloid formation during the labelling of carvedilol with ^99m^Tc is illustrated in Figure 7. The reaction was assessed over a pH range of 3–10 to determine optimal conditions for maximizing radiolabeling efficiency and purity. The optimal pH for achieving the highest radiochemical yield (96.5% ± 2.87%) was found to be 7. As pH increased to 7, the free technetium levels dropped sharply, indicating more effective reduction and binding at this neutral pH. The formation of colloidal tin was minimal at pH 7, as indicated by the low percentage of colloids (4%). The yield was maximized at a neutral pH (7), indicating that the reaction conditions at this pH were ideal for the efficient reduction of ^99m^TcO_4_^−^ and its complexation with carvedilol. This optimal condition balances the reduction of technetium and its complexation with carvedilol, thereby maximizing radiochemical yield while minimizing impurities. Deviations from this pH resulted in lower yields, as shown by the reduction in yield at both acidic and basic pH values. In more acidic conditions (pH below 7), the proportion of free ^99m^TcO_4_^−^ was significantly higher. This can be attributed to the incomplete reduction of ^99m^TcO_4_^−^ in highly acidic conditions, where the reducing agent (SnCl_2_) is less effective. The reducing power of Sn(II) was strongly pronounced in acidic media, promoting the reduction of technetium to a lower oxidation state suitable for [^99m^Tc]Tc-carvedilol formation, facilitating the reduction of technetium. However, excessive acidity hindered the complete binding of technetium to carvedilol, resulting in higher levels of free ^99m^TcO_4_^−^. In more alkaline conditions (pH above 7), the formation of reduced hydrolyzed technetium (colloidal tin) became more prevalent, particularly at pH 10. These colloids, which are radiochemical impurities, competed with carvedilol for the reduced technetium and did not contribute to the formation of the desired labeled compound. Therefore, maintaining the reaction mixture at a neutral pH (approximately 7) is critical for optimizing the labelling efficiency of carvedilol with ^99m^Tc, ensuring a high RCP and minimal formation of undesired byproducts.

### 3.5 Effect of concentration monitoring, biodistribution, and receptor occupancy studies

**FIGURE 8:**
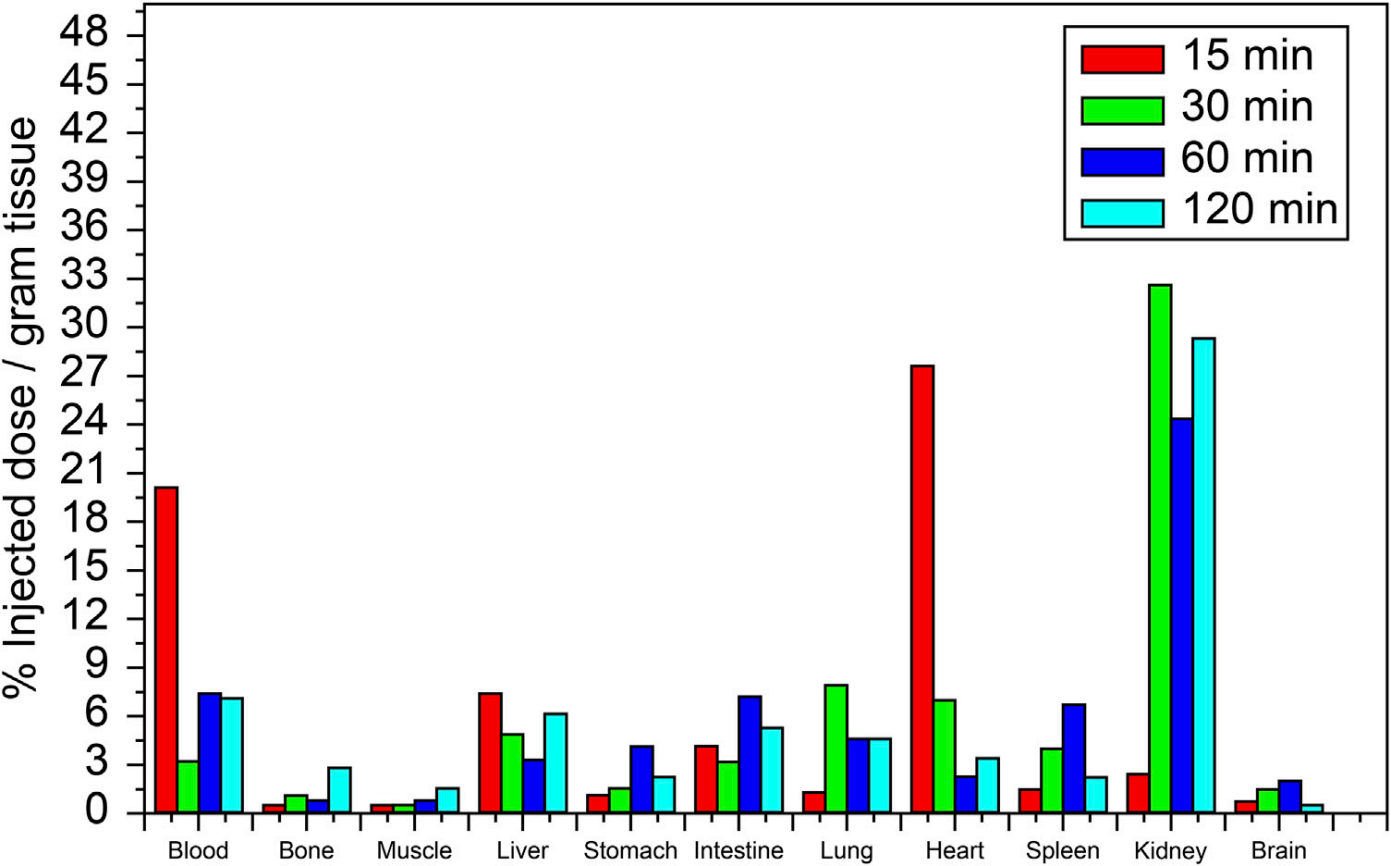
Biodistribution of the [^99m^Tc]Tc-carvedilol radioactivity complex in various organs of healthy albino mice at different time intervals (15, 30, 60, and 120 min). Each data point represents the mean percentage of injected dose per Gram of tissue (%ID/g) of four independent experiments conducted under standardized reaction conditions. For biodistribution-related measurements, error bars in the figure represent the SD from the mean uptake values (%ID/g) calculated for each group of six mice (six mice per experiment for each time point and organ), reflecting biological variability in variability in radiochemical yield, free pertechnetate, and colloid content across individual samples. Statistical analysis was conducted using a two-tailed Student’s t-test to compare uptake values between different time points and across selected organs. Significance was set at *P* ≤ 0.05.

The biodistribution profile of [^99m^Tc]Tc-carvedilol, detailing the mean % ID/g per organ ±SD and associated *P*-values for statistical significance, is provided in Table 1. The values are quantified as mean, with significant differences indicated by changes in the % ID/g organ ±SD of tissue at different time intervals. The metabolic and excretory pathways of [^99m^Tc]Tc-carvedilol are illustrated in Figure 8.

**TABLE 1:** *In vivo* biodistribution analyses of the [^99m^Tc]Tc-carvedilol in healthy Albino mice at different time intervals post-injection.

Tissues Organs	[^99m^Tc]Tc-carvedilol percentages of injected dose per Gram organ at different time intervals				Residual [^99m^Tc]Tc-carvedilol percentages of injected dose per Gram in non-targeted organs at different time intervals			
	15 min	30 min	60 min	120 min	15 min	30 min	60 min	120 min
Blood	19.895 ± 0.020^a^	3.969 ± 0.052	7.290 ± 0.052	5.972 ± 0.131	79.105 ± 0.05^a^	96.021 ± 0.01	92.710 ± 0.01	94.008 ± 0.02
Heart	27.533 ± 0.931^a^	7.273 ± 0.006	2.161 ± 0.006	3.516 ± 0.110	72.467 ± 0.02^a^	92.707 ± 0.02	97.838 ± 0.03	96.414 ± 0.07
Kidneys	2.764 ± 0.012	32.753 ± 0.345^a^	24.392 ± 0.345	29.284 ± 0.012	96.883 ± 0.07	67.207 ± 0.04^a^	75.608 ± 0.01	70.660 ± 0.05
Liver	7.255 ± 0.023	4.841 ± 0.568	3.325 ± 0.568	6.045 ± 1.020	92.740 ± 0.03	95.149 ± 0.01	95.109 ± 0.05	93.895 ± 0.04
Intestines	4.507 ± 0.521	3.358 ± 0.792	7.355 ± 0.792	5.347 ± 0.521	95.435 ± 0.09	96.612 ± 0.03	92.604 ± 0.04	94.643 ± 0.01
Stomach	1.193 ± 0.201	1.524 ± 0.259	4.263 ± 0.259	2.239 ± 0.065	98.807 ± 0.01	98.433 ± 0.04	95.706 ± 0.03	97.717 ± 0.06
Spleen	1.852 ± 0.151	4.174 ± 0.019	6.631 ± 0.019	2.279 ± 0.126	98.118 ± 0.02	95.816 ± 0.01	93.319 ± 0.05	97.701 ± 0.02
Muscle	0.671 ± 0.195	0.263 ± 0.215	0.659 ± 0.217	1.521 ± 0.201	99.309 ± 0.02	99.727 ± 0.01	99.321 ± 0.02	98.449 ± 0.03
Bone	0.322 ± 0.035	0.963 ± 0.215	0.675 ± 0.215	2.703 ± 0.019	99.638 ± 0.04	99.007 ± 0.03	99.304 ± 0.02	97.297 ± 0.01
Brain	0.631 ± 0.020	1.561 ± 0.008	2.895 ± 0.391	0.301 ± 0.110	99.359 ± 0.01	98.409 ± 0.03	97.103 ± 0.01	99.679 ± 0.02
Lung	1.290 ± 0.232	7.795 ± 0.060	4.590 ± 0.000	4.579 ± 0.111	98.710 ± 0.01	92.205 ± 0.01	95.410 ± 0.00	95.371 ± 0.04

The total sample size was 24 healthy Albino mice (mean of four experiments, six mice per experiment) at each time point and in each organ. Data points represent mean values from 24 healthy Albino mice per time point and per organ ±standard deviation.

^a^ Statistical analysis using Student’s t-test demonstrating a significant difference in mean % injected dose per Gram between groups, with significance set at *P* ≤ 0.05.

Vial content at pH 7; 100 µg carvedilol, 65 µg stannous chloride, and 1.0 mL (195 MBq) of ^99m^TcO_4_^−^; the reaction mixture was incubated at 25°C for 30 min.

At 15 min, the initial uptake of [^99m^Tc]Tc-carvedilol in the heart was remarkably high, peaking at 27.533 ± 0.931 % ID/g organ. This indicates a strong affinity and rapid selective localization of the radiopharmaceutical to the β_1_ adrenergic receptors of the cardiac tissue. Over the three intervals, the uptake exhibited a significant decline, stabilizing at a low but steady concentration, indicative of rapid perfusion and clearance from the heart. In contrast, the brain showed minimal but consistent uptake, peaking slightly at 2.895 ± 0.391 % ID/g organ at 60 min, indicating limited penetration across blood-brain barrier. The biodistribution findings further indicated efficient clearance of the complex from the bloodstream, with an initial high radioactivity level at 15 min (19.89 ± 0.020 % ID/g organ), followed by a rapid and significant decrease to 5.972 ± 0.131 % ID/g organ by 120 min. This pattern highlights the efficient distribution and systemic clearance of the radiopharmaceutical, accompanied by a moderate affinity for the alpha-1 adrenergic receptors, from the bloodstream over time.

The lungs peaked at 7.795 ± 0.060 % ID/g organ at 30 min and maintained a consistent concentration of 4.590 ± 0.000 % ID/g organ and 4.579 ± 0.111 % ID/g organ at 60 and 120 min, respectively, suggesting steady pulmonary distribution with a moderate affinity for β_2_ adrenergic receptors. Liver and kidney uptakes played significant roles in the metabolism and excretion of the compound, respectively. Initial hepatic uptake at 15 min was 7.255 ± 0.023 % ID/g organ, which increased to 7.355 ± 0.792 % ID/g organ at 60 min, reflecting its role in metabolism with a moderate rate of clearance. Notably, the kidneys exhibited the highest uptake across all time points, with values of 32.753 ± 0.345 % ID/g organ, 24.392 ± 0.345 % ID/g organ, and 29.284 ± 0.012 % ID/g organ at 30, 60, and 120 min, respectively. This was indicative of the kidneys being a major route of rapid filtration and primary excretion through the renal pathway.

Interestingly, the intestines demonstrated a marked increase from 3.358 ± 0.792 % ID/g organ at 30 min to a peak level of 7.355 ± 0.792 % ID/g organ at 60 min, before decreasing to 5.347 ± 0.521 at 120 min. This pattern indicates a complex interplay of initial accumulative absorption followed by secondary and gradual elimination processes through the hepatobiliary system. Muscle tissue showed a relatively low steady uptake of 0.671 ± 0.195 % ID/g organ, 0.263 ± 0.215 % ID/g organ, and 0.659 ± 0.217 % ID/g organ at 15, 30, and 60 min, respectively. Bone uptake was minimal at 30 min, with 0.963 ± 0.215 % ID/g organ, but slightly increased to 2.703 ± 0.019 % ID/g organ by 120 min. The sustained presence in the muscles and bones at later time points indicated some degree of nonspecific binding or slower clearance from these tissues. These results provide critical insights into the biodistribution profile and underscore the potential pharmacokinetic utility of [^99m^Tc]Tc-carvedilol in diagnostic cardiac imaging applications, while revealing its metabolic and excretory pathways.

Advancements in modern biotherapeutics for nuclear medicine (Nuclear medicine–advancing N) rely on the formulation of hypotheses regarding radiodiagnostic targets through a detailed understanding of the interplay between chemistry and biology. Biomolecules are strategically selected and conjugated with radioisotope markers to produce bioconjugates that ideally merge the molecular recognition capacity and biological activity of the biomolecule with the unique physicochemical properties of the radioisotopes. However, the overall functionality of these bioconjugates is governed by their complete molecular structure rather than by the isolated properties of their individual components. The radiolabeling process (Nuclear medicine–advancing N) therefore holds the potential to alter the biomolecule’s native activity or receptor specificity, possibly leading to diminished or lost biological function. To overcome these limitations, *in silico* molecular modeling and docking studies have been employed as predictive tools for assessing the structural and functional integrity of radiolabeled constructs. In this study, carvedilol derivatives (Dulin and Abraham, 2004)—known for their binding affinity to cardiac β_1_-adrenoceptors and their role in modulating adrenergic signaling—were explored through molecular modeling to generate an energy-optimized three-dimensional structure of [^99m^Tc]Tc-carvedilol (RCSB PDB) Subsequent docking simulations (Hsu et al., 2011) evaluated its interaction with β_1_-and β_2_-adrenoceptors to predict its binding specificity.

**FIGURE 9:**
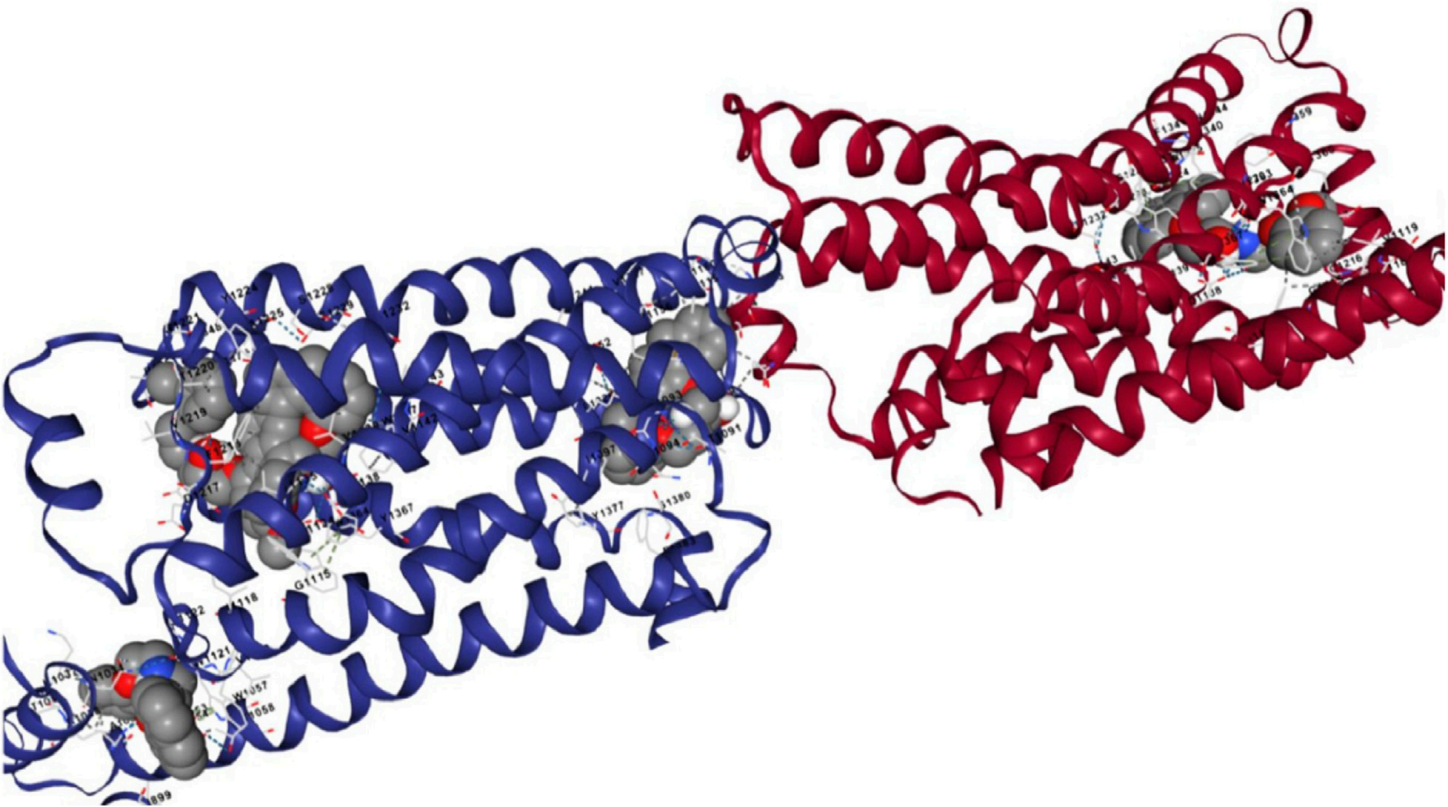
Interaction views of the molecular docking between [^99m^Tc]Tc-carvedilol and the myocardial adrenergic beta-1 receptor.

The docking analysis results, visualized in Figure 9, revealed that [^99m^Tc]Tc-carvedilol effectively occupied the active site cavities of β_1_-adrenoceptor, forming a stable complex through multiple non-covalent interactions. Among these, four hydrogen bonds played a central role in enhancing ligand-receptor stability, with key amino acid residues within the receptor’s binding pocket contributing to selective engagement and stabilization. These interactions were further supported by hydrophobic and electrostatic forces, which collectively ensured robust binding. The calculated binding energy of - 9.2 kcal/mol indicated a strong and thermodynamically favorable interaction, demonstrating that [^99m^Tc]Tc-carvedilol retained its biological activity and receptor specificity. This specificity was critical for targeting adrenergic receptors involved in myocardial function, particularly under pathological conditions, such as ischemia or heart failure. In-silico docking assessment underscored the structural stability and high binding affinity of the radiotracer, reinforcing its potential for nuclear medicine applications. These findings formed the basis for [^99m^Tc]Tc-carvedilol’s use as a non-invasive imaging agent for evaluating myocardial adrenergic activity, offering a promising tool for the diagnosis and monitoring of cardiovascular diseases.

**FIGURE 10:**
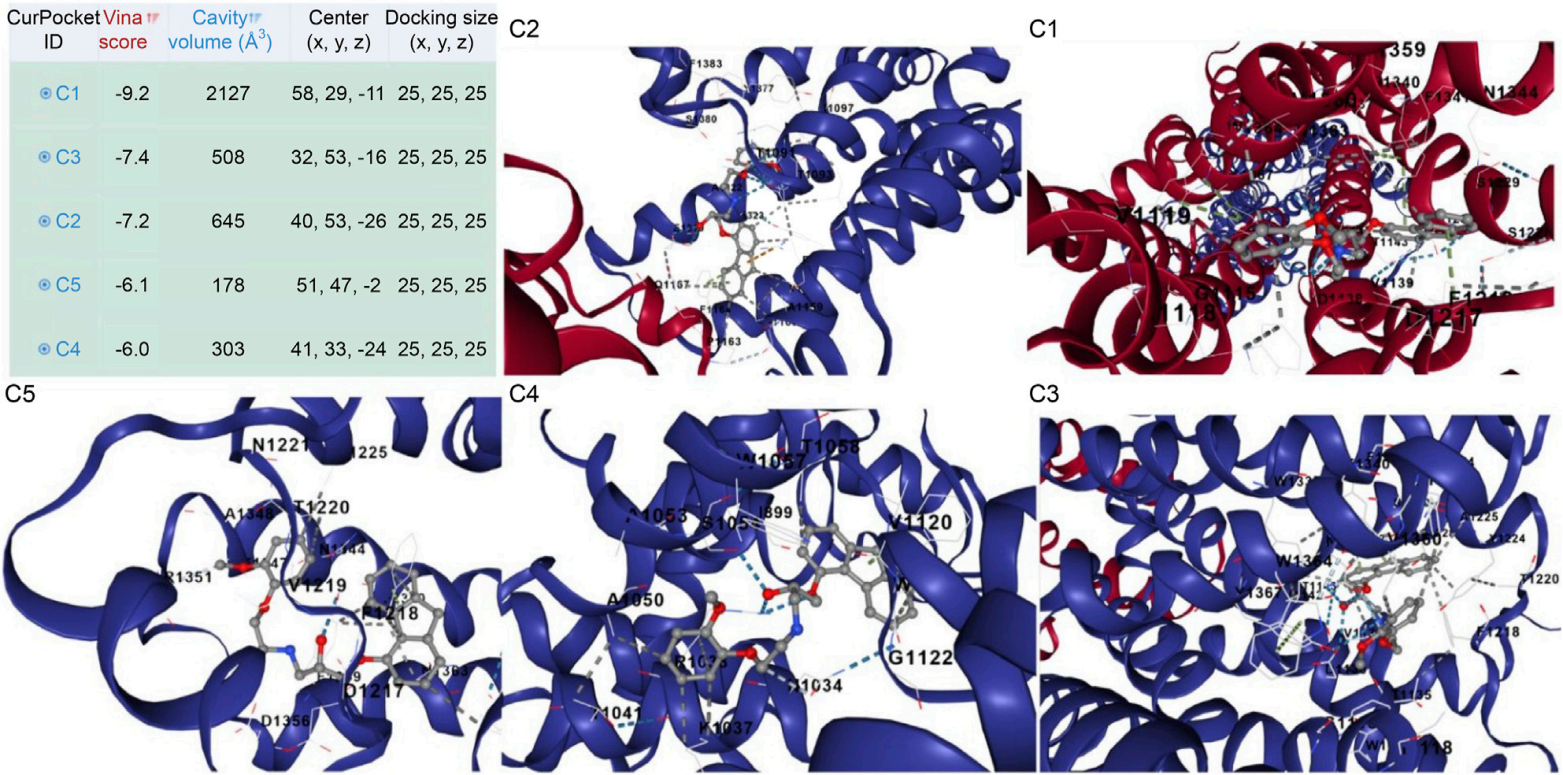
Protein structure of the [^99m^Tc]Tc-carvedilol and the myocardial adrenergic β_1_ receptor. The table presents results from molecular docking analysis, highlighting potential binding pockets (CurPocket IDs: C1 to C5) for a specific ligand-protein interaction. The corresponding figures (C1, C2, C3, C4, and C5) provide structural visualizations of the docking results, depicting the ligand’s interactions within each cavity. Key parameters include the cavity volume (Å^3^), which defines the physical space of each pocket, the center (x, y, z) representing the spatial coordinates of the pocket’s center, and the docking size (x, y, z) specifying the grid box dimensions utilized during docking simulations. Maroon-colored helices represent the secondary structure of the adrenergic β_1_ receptor, specifically its alpha helices. These structural elements are integral to the receptor’s conformation as a G-protein-coupled receptor. Their arrangement highlights the active site of the receptor where the interaction with [^99m^Tc]Tc-carvedilol occurs. Coloring enhances visibility and emphasizes the structural integrity of the protein. Gray atoms and bonds ([^99m^Tc]Tc-carvedilol) (C1): The ligand [^99m^Tc]Tc-carvedilol, is visualized as a set of interconnected gray atoms, showing its specific orientation within the binding pocket of the receptor. This positioning illustrates the molecular basis of its high affinity for receptors, which is crucial for its application in heart imaging. The dashed lines (binding interactions). Green dashed lines (C2) indicate hydrogen bonding or polar interactions between the ligand and specific residues of the receptor, reflecting the stabilization of the complex. Blue dashed lines (C3) likely denote ionic interactions or water-mediated bonding, which further enhances docking stability and specificity. Gray dashed lines (C4) indicate nonpolar or hydrophobic interactions contributing to the selectivity of the ligand. Highlighted residues (labeled amino acids) (C5): The labeled residues (e.g., D1217, F1218, C1216) mark the critical amino acids within the active site of the receptor that are directly involved in binding. These residues provide insights into the pharmacological and structural bases of the high specificity of [^99m^Tc]Tc-carvedilol for adrenergic β_1_ receptors.

The adrenergic β_1_ receptor structure, depicted by maroon-colored alpha helices, represented its secondary structure as a G-protein-coupled receptor, emphasizing the integrity and conformation of its active site for ligand interactions, as shown in Figure 10. The Vina docking score (in kcal/mol) represents the ligand-protein binding free energy, accounting for van der Waals interactions, electrostatic forces, hydrogen bonding, and other molecular contacts. The ligand [^99m^Tc]Tc-carvedilol, visualized as interconnected gray atoms, was precisely positioned within the binding pocket of the receptor, demonstrating its high binding affinity (largest cavity volume (2127 Å^3^) and strong Vina score of −9.2 kcal/mol), which is crucial for heart imaging applications (Figure 10. C1). The various dashed lines highlight the molecular interactions stabilizing the ligand-receptor complex; green indicates hydrogen or polar bonds (Figure 10. C2), blue represents ionic or water-mediated interactions (Figure 10. C3), and gray indicates hydrophobic interactions (Figure 10. C4), all of which contribute to docking stability and specificity. Key amino acid residues within the receptor’s active site (e.g., D1217, F1218, V1219) were labeled (Figure 10. C5), underscoring their critical roles in the pharmacological properties of the receptor and the selective binding of [^99m^Tc]Tc-carvedilol. These structural insights enhance the understanding of the receptor-ligand dynamics, which is essential for radiodiagnostic applications.

## 4 Discussion

This study significantly advances the field of cardiovascular receptor imaging by introducing [^99m^Tc]Tc-carvedilol as a novel radiopharmaceutical specifically engineered for the non-invasive quantification of cardiac adrenergic receptor binding sites. The molecular design of [^99m^Tc]Tc-carvedilol is strategically based on carvedilol’s high intrinsic affinity for β_1_-, β_2_-, and α_1_-adrenergic receptors—key regulators of myocardial contractility and pathological remodeling (Dulin and Abraham, 2004). The parent molecule’s structure offers multiple electron-donating groups, including hydroxyl, amine, and ether moieties, which serve as coordination sites for radiolabeling. Radiolabeling carvedilol with technetium-99m, a widely utilized gamma-emitting radionuclide known for its optimal physical and imaging properties, enabled the development of a stable [^99m^Tc]Tc-carvedilol complex through direct chelation. The coordination environment effectively stabilizes the radionuclide while preserving the receptor-binding activity of carvedilol, facilitating selective myocardial targeting. This chelation strategy mirrors established methodologies for technetium-based tracers that aim to retain pharmacological specificity while optimizing biodistribution and *in vivo* stability. The resulting radiotracer demonstrated several advantageous properties: selective myocardial uptake, rapid systemic clearance, minimal non-target organ accumulation, and robust *in vivo* stability, all of which contribute to high-resolution adrenergic receptor mapping. Importantly, optimization of the radiolabeling protocol using SnCl_2_·2H_2_O as a reducing agent ensured consistently high labeling efficiency with minimal radiochemical impurities, enhancing the compound’s diagnostic reliability. Biodistribution studies further validated the approach, confirming strong myocardial affinity with negligible accumulation in the brain and other non-target tissues, thereby reducing background signal and ensuring high-fidelity imaging performance (Wells et al., 2011).

Radiochemical stability studies further validated the compound’s robustness, with the [^99m^Tc]Tc-carvedilol complex maintaining structural integrity and high radiopurity in human serum over 24 h—a feature essential for clinical translation, consistent with previous evidence on the importance of tracer stability in dynamic physiological conditions (Naqvi et al., 2018). When benchmarked against earlier agents such as iodine-131-labeled carvedilol (Motaleb et al., 2023), limited by slow clearance, low receptor specificity, and extended imaging times [^99m^Tc]Tc-carvedilol demonstrates superior pharmacokinetics. The shorter half-life of technetium-99m not only reduces radiation burden but also facilitates timely image acquisition, aligning well with the demands of acute and emergency cardiovascular assessments. Furthermore, the intrinsic imaging properties of technetium-99m, including its optimal gamma emission energy and favorable biological behavior when coupled with stable chelation techniques, significantly enhance tracer performance in imaging models (Khan et al., 2019). These integrated properties facilitate precise mapping of adrenergic receptor distribution within cardiac tissue, a critical advancement for enhancing image resolution and diagnostic specificity in clinical cardiology.

Optimization of the radiolabeling protocol, through systematic modulation of carvedilol concentration, reducing agent content, reaction time, and pH, resulted in consistently high labeling efficiency. This translates into rapid and reliable myocardial uptake within the critical 15-min post-injection window, enhancing its utility for time-sensitive diagnostics. Furthermore, the compound’s minimal degradation under physiological pH supports efficient systemic clearance from non-target tissues while preserving myocardial signal integrity. Importantly, this study addresses limitations associated with existing technetium-based radiotracers, such as ^99m^Tc-sestamibi (Manabe et al., 2018) and ^99m^Tc-tetrofosmin (Zaret et al., 1995). Both agents are constrained by non-specific uptake and hepatobiliary excretion, which contribute to persistent background activity that compromises image clarity—particularly near the heart. In contrast [^99m^Tc]Tc-carvedilol exhibits lower off-target accumulation and improved clearance kinetics, offering a more refined imaging profile. Collectively, these features position [^99m^Tc]Tc-carvedilol as a promising next-generation diagnostic radiopharmaceutical with a potential for enhancing the precision of cardiovascular disease evaluation.

Crucially [^99m^Tc]Tc-carvedilol not only enables high-resolution visualization of cardiac adrenergic receptors but also facilitates longitudinal tracking of diagnostic indicators, establishing its value across both acute and chronic cardiovascular disease settings. When compared to PET radiotracers labeled with fluorine-18—such as 1′-[^18^F]fluorometoprolol (de Groot et al., 1993), which has shown promise for receptor-specific cardiac imaging—technetium-99m-based SPECT agents offer notable practical and clinical advantages. Despite the high sensitivity of PET imaging, fluorine-18 tracers (Lu et al., 2024) are limited by their short half-life, complex synthesis processes, elevated operational costs, and limited accessibility in many clinical settings. In contrast, ^99m^Tc-labeled compounds benefit from simpler radiolabeling protocols, widespread availability, and lower overall healthcare costs without sacrificing diagnostic accuracy (Naqvi, 2022). Moreover, the favorable physical properties of technetium-99m, including its optimal photon energy (Beller and Zaret, 2000) and minimal redistribution (Duatti, 2001), support precise imaging with reduced radiation exposure. These attributes not only enhance image quality and diagnostic consistency but also make [^99m^Tc]Tc-carvedilol a more scalable and clinically accessible platform for real-time cardiovascular assessment.

### 4.1 Implications for future research

As demand for targeted nuclear tracers increases [^99m^Tc]Tc-carvedilol stands out as a versatile agent for both receptor mapping and functional evaluation of therapies. The clinical relevance of [^99m^Tc]Tc-carvedilol is further underscored by its alignment with known pathophysiological dynamics of adrenergic receptor subtypes. In cardiac tissue (Santos and Spadari-Bratfisch, 2006), β_1_ receptors are integral to regulating contractility and heart rate; however, in conditions such as heart failure or post-myocardial infarction, these receptors undergo downregulation (Lymperopoulos et al., 2013), while β_2_ receptors may become pathologically upregulated contributing to maladaptive remodeling and arrhythmogenesis (Lymperopoulos et al., 2013). Carvedilol, with its antagonistic affinity for β_1_, β_2_, and α_1_ receptors, provides a mechanistic basis for both imaging and intervention (D and argie, 2001). Its pharmacological effects, including reduction in myocardial oxygen demand, blood pressure control, and attenuation of sympathetic overdrive, are consistent with the goals of receptor-guided diagnostic strategies. In both experimental and clinical contexts, carvedilol has demonstrated cardioprotective benefits (Chen-Scarabelli et al., 2012) without provoking excessive bradycardia or hypotension, making it particularly suitable for emergency use cases such as hypertensive crises and acute coronary syndromes. These functionalities enable clinicians to tailor treatments based on individual receptor profiles, thereby advancing the paradigm of precision medicine in cardiovascular care (Pellico et al., 2021). Future studies exploring its application in receptor-guided dosing strategies could pave the way for more personalized treatment protocols (Graves and Hobbs, 2021) that optimize efficacy while reducing systemic exposure.

Technetium-99m has emerged as a versatile radiolabeling agent for developing targeted radiotracers, with robust clinical evidence supporting its efficacy in cardiovascular and cerebrovascular diagnostics (Azhari and Hashem, 2024) and its precision in stroke applications (Azhari, 2024). When paired with ongoing innovations in ligand design and advanced imaging technologies, such as high-sensitivity detectors, dual-isotope techniques (Hijnen et al., 2012), and hybrid modalities, its clinical utility is expected to expand, particularly in complex scenarios like post-MI stroke, where concurrent assessment of perfusion and receptor status is essential. These integrated advancements, especially when combined with the molecular targeting capabilities of [^99m^Tc]Tc-carvedilol, have the potential to reshape diagnostic strategies in nuclear cardiology.

### 4.2 Strengths and limitations

This study presents a significant advancement in nuclear cardiology by introducing [^99m^Tc]Tc-carvedilol as a novel, selectively targeted radioligand for non-invasive myocardial receptor assessment. Through rigorous radiolabeling optimization and comprehensive biodistribution analysis, the research demonstrates the tracer’s strong cardiac affinity, high radiochemical stability, and translational feasibility. The integration of *in vivo* pharmacokinetic profiling with molecular docking further substantiates its receptor-specific binding, reinforcing its diagnostic precision. By bridging molecular pharmacology with clinical applications, this work lays a critical foundation for the development of targeted, reproducible, and clinically adaptable radiopharmaceuticals in cardiovascular diagnostics.

The rationale for selecting a 24 h incubation period in the *in vitro* stability study is grounded in radiopharmaceutical best practices, where extended incubation simulates worst-case conditions for potential degradation. Although the physical half-life of ^99m^Tc is approximately 6 h, a 24 h time frame enables the observation of radiolytic breakdown, transchelation events, or colloidal formation that may occur over the useful diagnostic window post-injection. This approach ensures that the compound maintains radiochemical integrity even under prolonged exposure, which is particularly important when considering logistical delays or extended imaging protocols in clinical settings. Moreover, the 24 h assessment serves as a benchmark for comparing the tracer’s stability profile with other technetium-labeled compounds used in cardiac imaging and ensures compliance with international radiopharmaceutical standards (Gillings et al., 2021). The observed radiochemical stability throughout this period supports the tracer’s reliability for biodistribution and imaging applications, even beyond the typical *in vivo* circulation timeframe, thereby validating its translational potential for clinical use.

While biodistribution studies provides quantitative insight into the anatomical localization and temporal kinetics of radiotracer uptake, it does not distinguish between non-specific accumulation and receptor-mediated binding. Therefore, the receptor occupancy study was incorporated to validate the functional targeting capability of the radiotracer, confirming that cardiac uptake is driven by specific ligand-receptor interactions rather than by perfusion alone or nonspecific binding. This approach is especially critical in the context of carvedilol—a non-selective β-blocker with high β_1_-adrenergic receptor affinity—where receptor specificity underpins its clinical utility in cardiac imaging and pharmacological profiling (Joshi et al., 2024). The receptor occupancy study employed competitive displacement using excess unlabeled carvedilol to quantify the proportion of radiotracer specifically bound to receptors *versus* that retained non-specifically. This quantification is essential for determining binding potential, calculating receptor density, and refining kinetic modeling parameters for potential SPECT imaging applications.

While *in vitro* serum-based assays offer valuable preliminary insight into the structural and radiochemical stability of radiolabeled compounds, they inherently lack the complexity and physiological dynamics of living systems. Accordingly, to enhance the translational validity of the findings, this study incorporated *in vivo* biodistribution and receptor occupancy analyses using albino rat models. It is important to clarify that the study did not aim to establish direct equivalence between human and rat serum environments. Rather, each model was employed to answer distinct yet complementary research questions: human serum assays assessed the physicochemical integrity of [^99m^Tc]Tc-carvedilol in a clinically relevant matrix, while *in vivo* models were used to investigate systemic distribution and myocardial receptor targeting under biological conditions. This dual-method approach exemplifies a stepwise translational research strategy that aligns with pre-clinical development standards and enhances scientific rigor. Although preclinical data demonstrated promising myocardial uptake and target engagement, physiological differences between animal models and humans must be acknowledged, as they may influence biodistribution patterns and receptor affinity. Furthermore, the use of healthy animal models, while necessary for initial validation, may not fully capture the altered pharmacokinetics present in pathological cardiovascular conditions—necessitating future studies in disease-specific models.

In this study, molecular docking was employed as an initial, hypothesis-generating strategy to explore potential binding modes, key interaction patterns, and energetically favorable conformations between [^99m^Tc]Tc-carvedilol and the β_1_-adrenoceptor. While geometry optimization, energy minimization, and careful ligand-receptor preparation were rigorously conducted to ensure docking reliability, it is recognized that molecular docking provides theoretical predictions rather than definitive proof of stable binding. Full validation through molecular dynamics simulations, followed by binding free energy calculations using Molecular Mechanics Poisson–Boltzmann Surface Area or Molecular Mechanics Generalized Born Surface Area methods, will be essential in future studies to confirm binding stability and to quantify dynamic interaction energies.

Although the relatively short half-life of technetium-99m is advantageous for minimizing patient radiation exposure, it may limit the temporal resolution of extended imaging protocols. Therefore, future research should prioritize clinical trials to confirm these findings in human subjects and determine optimal imaging windows that maximize diagnostic efficacy. This study provides a foundational framework for the development of [^99m^Tc]Tc-carvedilol as a selective myocardial radioligand, with significant potential to address the critical need for high-specificity agents in nuclear cardiology.

### 4.3 Conclusion

This study contributes to the evolving landscape of nuclear cardiology by introducing [^99m^Tc]Tc-carvedilol as a novel radiopharmaceutical candidate with the potential to transform myocardial imaging through selective targeting of cardiac β_1_-adrenoceptors. Rather than simply adding to the inventory of radiotracers, this work emphasizes a precision-driven approach in radiopharmaceutical design—merging molecular modeling, radiolabeling chemistry, and *in vivo* biodistribution analysis to develop agents with enhanced target specificity, metabolic stability, and clinical translatability.

The strategic integration of computational and biological methods in this study supports a paradigm shift from empirical tracer development toward mechanism-informed design, with [^99m^Tc]Tc-carvedilol exemplifying this transition. Its performance in preclinical models, characterized by selective myocardial uptake and favorable clearance kinetics, underscores its potential to enhance diagnostic accuracy and facilitate receptor quantification, thereby contributing to more refined diagnostic stratification in cardiovascular diseases. This work also underscores the translational pathway yet to be completed. While the preclinical data are encouraging, the compound’s diagnostic value and safety profile must be rigorously evaluated in clinical trials across diverse patient populations and disease states. Comparative assessments against standard tracers will be essential to demonstrate added value in real-world scenarios, especially in pathologies such as ischemic heart disease, heart failure, and receptor remodeling syndromes.

In summary, the development of [^99m^Tc]Tc-carvedilol marks an important step toward a new generation of cardiac imaging agents designed with both molecular precision and clinical utility in mind. This study lays a critical foundation for future investigations that bridge the gap between bench and bedside, ultimately advancing personalized diagnostics and targeted care in cardiovascular medicine.

## Data Availability

The original contributions presented in the study are included in the article/supplementary material, further inquiries can be directed to the corresponding author.

## References

[B1] Abdu Hussen A. A. (2022). High-performance liquid chromatography (HPLC): a review. Ann. Adv. Chem. 6 (1), 010–020. 10.29328/journal.aac.1001026

[B2] Ajoolabady A. Pratico D. Ren J. (2024). Endothelial dysfunction: mechanisms and contribution to diseases. Acta Pharmacol. Sin. 45 (10), 2023–2031. 10.1038/s41401-024-01295-8 38773228 PMC11420364

[B3] Al Oraini D. (2018). Calibration of the absolute efficiency of well-type NaI(Tl) scintillation detector in 0.121–1.408 MeV energy range. Sci. Technol. Nucl. Install. 2018, 1–6. 10.1155/2018/6432380

[B4] Azhari H. F. (2024). Advancing stroke diagnosis and management through nuclear medicine: a systematic review of clinical trials. Front. Med. 19 (11), 1425965. 10.3389/fmed.2024.1425965 PMC11368133 39224610

[B5] Azhari H. F. Hashem A. M. (2024). Quantifying AMPARs with ^99m^Tc-omberacetam: a novel diagnostic radiotracer for ischemic stroke. JUmm Al-Qura Univ. Appll Sci. 10 (1), 211–224. 10.1007/s43994-023-00093-y

[B6] Beattie K. Phadke G. Novakovic J. (2013). Carvedilol. Profiles Drug Subst. Excip. Relat. Methodol. 38, 113–157. 10.1016/B978-0-12-407691-4.00004-6 23668404

[B7] Beller G. A. Zaret B. L. (2000). Contributions of nuclear cardiology to diagnosis and prognosis of patients with coronary artery disease. Circulation 101 (12), 1465–1478. 10.1161/01.cir.101.12.1465 10736294

[B8] Boschi A. Uccelli L. Marvelli L. Cittanti C. Giganti M. Martini P. (2022). Technetium-99m radiopharmaceuticals for ideal myocardial perfusion imaging: lost and found opportunities. Molecules 27 (4), 1188. 10.3390/molecules27041188 35208982 PMC8877792

[B9] Bristow M. R. (2000). beta-adrenergic receptor blockade in chronic heart failure. Circulation 101 (5), 558–569. 10.1161/01.CIR.101.5.558 10662755

[B10] ChemAxon Cheminformatics software for the next generation of scientists. Available online at: https://chemaxon.com/ (Accessed May 28, 2025).

[B11] Chen Z. Venkat P. Seyfried D. Chopp M. Yan T. Chen J. (2017). Brain-heart interaction: cardiac complications after stroke. Circ. Res. 121 (4), 451–468. 10.1161/CIRCRESAHA.117.311170 28775014 PMC5553569

[B12] Chen-Scarabelli C. Saravolatz L. Murad Y. Shieh W. S. Qureshi W. Di Rezze J. (2012). A critical review of the use of carvedilol in ischemic heart disease. Am. J. Cardiovasc. Drugs 12 (6), 391–401. 10.1007/BF03262473 23061698

[B13] Cuocolo A. Cittanti C. Acampa W. Larobina M. Petretta M. (2011). Current and future status of blood flow tracers. Curr. Cardiovasc. Imaging Rep. 4 (3), 227–236. 10.1007/s12410-011-9081-9

[B14] Dargie H. J. (2001). Effect of carvedilol on outcome after myocardial infarction in patients with left-ventricular dysfunction: the capricorn randomised trial. Lancet 357 (9266), 1385–1390. 10.1016/S0140-6736(00)04560-8 11356434

[B15] de Groot T. J. van Waarde A. Elsinga P. H. Visser G. M. Brodde O. E. Vaalburg W. (1993). Synthesis and evaluation of 1′-[^18^F]fluorometoprolol as a potential tracer for the visualization of β-adrenoceptors with PET. Nucl. Med. Biol. 20 (5), 637–642. 10.1016/0969-8051(93)90033-q 8395276

[B16] Dobrucki L. W. Sinusas A. J. (2010). PET and SPECT in cardiovascular molecular imaging. Nat. Rev. Cardiol. 7 (1), 38–47. 10.1038/nrcardio.2009.201 19935740

[B17] Duatti A. (2001). Review on ^99m^Tc radiopharmaceuticals with emphasis on new advancements. Nucl. Med. Biol. 92, 202–216. 10.1016/j.nucmedbio.2020.05.005 32475681

[B18] Duffield S. Da Vià L. Bellman A. C. Chiti F. (2021). Automated high-throughput partition coefficient determination with image analysis for rapid reaction workup process development and modeling. Org. Process Res. Dev. 25 (12), 2738–2746. 10.1021/acs.oprd.1c00343

[B19] Dulin B. Abraham W. T. (2004). Pharmacology of carvedilol. Am. J. Cardiol. 93 (9A), 3B–6B. 10.1016/j.amjcard.2004.01.003 15144929

[B20] Gardner R. T. Ripplinger C. M. Myles R. C. Habecker B. A. (2016). Molecular mechanisms of sympathetic remodeling and arrhythmias. Circ. Arrhythm. Electrophysiol. 9 (2), e001359. 10.1161/CIRCEP.115.001359 26810594 PMC4730917

[B21] Gillings N. Hjelstuen O. Ballinger J. Behe M. Decristoforo C. Elsinga P. (2021). Guideline on current good radiopharmacy practice (cGRPP) for the small-scale preparation of radiopharmaceuticals. EJNMMI Radiopharm. Chem. 6 (1), 8. 10.1186/s41181-021-00123-2 33580358 PMC7881071

[B22] Graves S. A. Hobbs R. F. (2021). Dosimetry for optimized, personalized radiopharmaceutical therapy. Semin. Radiat. Oncol. 31 (1), 37–44. 10.1016/j.semradonc.2020.07.008 33246635

[B23] Hijnen N. M. de Vries A. Nicolay K. Grüll H. (2012). Dual-isotope 111In/177Lu SPECT imaging as a tool in molecular imaging tracer design. Contrast Media Mol. Imaging 7 (2), 214–222. 10.1002/cmmi.485 22434634

[B24] Hsu K. C. Chen Y. F. Lin S. R. Yang J. M. (2011). iGEMDOCK: a graphical environment of enhancing GEMDOCK using pharmacological interactions and post-screening analysis. BMC Bioinforma. 12 (Suppl. 1), S33. 10.1186/1471-2105-12-S1-S33 PMC3044289 21342564

[B25] IAEA (2021). Country nuclear power profiles; Egypt. Available online at: https://www-pub.iaea.org/MTCD/Publications/PDF/CNPP-2021/countryprofiles/Egypt/Egypt.htm (Accessed May 28, 2025).

[B26] Joshi S. S. Geers J. Gimelli A. Hyafil F. Habib G. Erba P. (2024). Current and emerging radiotracers in molecular cardiovascular imaging. Circ. Cardiovasc. Imaging 17, e016323. 10.1161/CIRCIMAGING.123.016323 39405389

[B27] Khan N. U. H. Naqvi S. A. R. Roohi S. Sherazi T. A. Khan Z. A. Zahoor A. F. (2019). Technetium-99m radiolabeling and biological study of epirubicin for *in vivo* imaging of multi-drug-resistant *Staphylococcus aureus* infections *via* single photon emission computed tomography. Chem. Biol. Drug Des. 93 (2), 154–162. 10.1111/cbdd.13393 30216686

[B28] Kirkwood J. (2013). AVMA guidelines for the euthanasia of animals. Anim. Welf. 22 (3), 412. 10.1017/S0962728600005492

[B29] Kumari V. B. C. Patil S. M. Ramu R. Shirahatti P. S. Kumar N. Sowmya B. P. (2022). “Chapter 5. Chromatographic techniques: types, principles, and applications,” in Analytical techniques in biosciences. Editors Egbuna C. Patrick-Iwuanyanwu K. C. Shah M. A. Ifemeje J. C. Rasul A. (India: Academic Press), 73–101. 10.1016/B978-0-12-822654-4.00013-0

[B30] Lu X. Deng X. Kong F. Hai W. Luo Q. (2024). Construction and preliminary evaluation of two ^18^F-labeled radiopharmaceuticals for myocardial perfusion imaging. Anal. Chem. 96 (29), 11725–11733. 10.1021/acs.analchem.4c00709 38975941

[B31] Lymperopoulos A. Rengo G. Koch W. J. (2013). Adrenergic nervous system in heart failure: pathophysiology and therapy. Circ. Res. 113 (6), 739–753. 10.1161/CIRCRESAHA.113.300308 23989716 PMC3843360

[B32] Maioli C. Luciniani G. Strinchini A. Tagliabue L. Del Sole A. (2017). Quality control on radiochemical purity in Technetium-99m radiopharmaceuticals labelling: three years of experience on 2280 procedures. Acta Biomed. 88 (1), 49–56. 10.23750/abm.v88i1.5285 28467334 PMC6166199

[B33] Manabe O. Kikuchi T. Scholte A. J. H. A. El Mahdiui M. Nishii R. Zhang M. R. (2018). Radiopharmaceutical tracers for cardiac imaging. J. Nucl. Cardiol. 25 (4), 1204–1236. 10.1007/s12350-017-1131-5 29196910 PMC6133155

[B34] Martini P. Boschi A. Cicoria C. Uccelli L. Pasquali M. Duatti A. (2016). A solvent-extraction module for cyclotron production of high-purity technetium-99m. Appl. Radiat. Isotopes 118, 302–307. 10.1016/j.apradiso.2016.10.002 27744212

[B35] Mercado R. Lagos S. Velásquez E. (2023). “Radiochemical purity and identity in radiopharmaceuticals: design and improvement of quality control methods by HPLC,” in Advances in dosimetry and new trends in radiopharmaceuticals. Editors Adelaja Osibote O. Eppard E. (Rijeka, Croatia: IntechOpen). 10.5772/intechopen.112355

[B36] Molavipordanjani S. Tolmachev V. Hosseinimehr S. J. (2019). Basic and practical concepts of radiopharmaceutical purification methods. Drug Discov. Today 24 (1), 315–324. 10.1016/j.drudis.2018.09.018 30278224

[B37] Motaleb M. A. Attalah K. M. Shweeta H. A. Ibrahim I. T. (2023). Synthesis and biological evaluation of [^131^i]iodocarvedilol as a potential radiopharmaceutical for heart imaging. BMC Chem. 17 (1), 21. 10.1186/s13065-023-00935-0 36922888 PMC10018969

[B38] Mourouzis K. Oikonomou E. Siasos G. Tsalamadris S. Vogiatzi G. Antonopoulos A. (2020). Pro-inflammatory cytokines in acute coronary syndromes. Curr. Pharm. Des. 26 (36), 4624–4647. 10.2174/1381612826666200413082353 32282296

[B39] Naqvi S. A. Matzow T. Finucane C. Nagra S. A. Ishfaq M. M. Mather S. J. (2010). Insertion of a lysosomal enzyme cleavage site into the sequence of a radiolabeled neuropeptide influences cell trafficking *in vitro* and *in vivo* . Cancer Biother Radiopharm. 25 (1), 89–95. 10.1089/cbr.2009.0666 20187801

[B40] Naqvi S. A. R. (2022). ^99m^ Tc-labeled antibiotics for infection diagnosis: mechanism, action, and progress. Chem. Biol. Drug Des. 99 (1), 56–74. 10.1111/cbdd.13923 34265177

[B41] Naqvi S. A. R. Drlica K. (2017). Fluoroquinolones as imaging agents for bacterial infection. Dalton Trans. 46 (42), 14452–14460. 10.1039/c7dt01189j 28920628 PMC5739321

[B42] Naqvi S. A. R. Roohi S. Iqbal A. Sherazi T. A. Zahoor A. F. Imran M. (2018). Ciprofloxacin: from infection therapy to molecular imaging. Mol. Biol. Rep. 45 (5), 1457–1468. 10.1007/s11033-018-4220-x 29974398

[B43] National Institute of Health (2024). The institutional animal care and use committee. Available online at: https://olaw.nih.gov/resources/tutorial/iacuc.htm (Accessed May 28, 2025).

[B44] Nuclear Medicine – Advancing N. Medicine through innovation – NCBI Bookshelf. Available online at: https://www.ncbi.nlm.nih.gov/books/NBK11471/uclear (Accessed May 28, 2025).

[B45] Owen D. B. (1965). The power of Student’s *t*-test. J. Am. Stat. Assoc. 60 (309), 320–333. 10.1080/01621459.1965.10480794

[B46] Palasubramaniam J. Wang X. Peter K. (2019). Myocardial infarction-from atherosclerosis to thrombosis. Arterioscler. Thromb. Vasc. Biol. 39 (8), e176–e185. 10.1161/ATVBAHA.119.312578 31339782

[B47] Pellico J. Gawne P. J. T M de Rosales R. T. M. (2021). Radiolabelling of nanomaterials for medical imaging and therapy. Chem. Soc. Rev. 50 (5), 3355–3423. 10.1039/d0cs00384k 33491714

[B48] Percie du Sert N. P. Hurst V. Ahluwalia A. Alam S. Avey M. T. Baker M. (2020). The ARRIVE guidelines 2.0: updated guidelines for reporting animal research. PLOS Biol. 18 (7), e3000410. 10.1371/journal.pbio.3000410 32663219 PMC7360023

[B49] Putaala J. Nieminen T. (2018). Stroke risk period after acute myocardial infarction revised. J. Am. Heart Assoc. 7 (22), e011200. 10.1161/JAHA.118.011200 30571506 PMC6404430

[B50] Rathmann S. M. Ahmad Z. Slikboer S. Bilton H. A. Snider D. P. Valliant J. F. (2019). “The radiopharmaceutical chemistry of technetium-99m,” in Radiopharmaceutical chemistry. Editors Lewis J. S. Windhorst A. D. Zeglis B. M. (Cham: Springer), 311–333. 10.1007/978-3-319-98947-1_18

[B51] RCSB PDB. Available online at: https://www.rcsb.org/ (Accessed May 28, 2025).

[B52] Santos I. N. Spadari-Bratfisch R. C. (2006). Stress and cardiac beta adrenoceptors. Stress 9 (2), 69–84. 10.1080/10253890600771858 16895831

[B53] Serdar C. C. Cihan M. Yücel D. Serdar M. A. (2021). Sample size, power and effect size revisited: simplified and practical approaches in pre-clinical, clinical and laboratory studies. Biochem. Med. Zagreb. 31 (1), 010502. 10.11613/BM.2021.010502 33380887 PMC7745163

[B54] Shimadzu Corporation. Software download. Available online at: https://www.shimadzu.com/an/products/gas-chromatography/gc-accessories-components/advanced-flow-technology-series/sw-dl/index.html (Accessed May 28, 2025).

[B55] Singhal A. Saini U. Chopra B. Dhingra A. K. Jain A. Chaudhary J. (2024). UV-visible spectroscopy: a review on its pharmaceutical and bio-allied sciences applications. Curr. Pharm. Anal. 20 (3), 161–177. 10.2174/0115734129300562240408042614

[B56] Smith A. Mancini M. Nie S. (2009). Second window for *in vivo* imaging. Nat. Nanotech 4, 710–711. 10.1038/nnano.2009.326 PMC2862008 19898521

[B57] Software.informer (2014). CambridgeSoft ChemBioOffice. Available online at: https://cambridgesoft-chembiooffice-2014.software.informer.com/14.0/.

[B58] Sposato L. A. Hilz M. J. Aspberg S. Murthy S. B. Bahit M. C. Hsieh C. Y. (2020). Post-stroke cardiovascular complications and neurogenic cardiac injury: JACC state-of-the-art review. J. Am. Coll. Cardiol. 76 (23), 2768–2785. 10.1016/j.jacc.2020.10.009 33272372

[B59] Strosberg A. D. (1993). Structure, function, and regulation of adrenergic receptors. Protein Sci. 2 (8), 1198–1209. 10.1002/pro.5560020802 8401205 PMC2142449

[B60] Tsao C. W. Aday A. W. Almarzooq Z. I. Alonso A. Beaton A. Z. Bittencourt M. S. (2022). Heart disease and stroke Statistics-2022 update: a report from the American heart association. Circulation 145 (8), 153–e639. 10.1161/CIR.0000000000001052 35078371

[B61] Ume C. S. Erdoğan A. (2012). Reaction kinetics of carbon dioxide with 2-amino-2-hydroxymethyl-1,3-propanediol in aqueous solution obtained from the stopped flow method. Turkish J. Chem. 36 (3), 6. 10.3906/kim-1107-33

[B62] Usmani S. Rasheed R. Al Kandari F. Marafi F. Naqvi S. A. R. (2019). 225Ac prostate-specific membrane antigen posttherapy α imaging: comparing 2 and 3 photopeaks. Clin. Nucl. Med. 44 (5), 401–403. 10.1097/RLU.0000000000002525 30932973

[B63] Wells G. Prest H. Russ C. W. (2011). Signal, noise, and detection limits in mass spectrometry. Technical Note for Agilent Technologies Inc.

[B64] Winkler G. Wolschann P. Briza P. Heinz F. X. Kunz C. (1985). Spectral properties of trifluoroacetic Acid—Acetonitrile gradient systems for separation of picomole quantities of peptides by reversed-phase high-performance liquid chromatography. J. Chromatogr. A 347, 83–88. 10.1016/S0021-9673(01)95471-8

[B65] Zaret B. L. Rigo P. Wackers F. J. T. Hendel R. C. Braat S. H. Iskandrian A. S. (1995). Myocardial perfusion imaging with ^99m^Tc-tetrofosmin: comparison to ^201^Tl imaging and coronary angiography in a phase III multicenter trial. Circulation 91 (2), 313–319. 10.1161/01.cir.91.2.313 7805233

[B66] Zhang H. Yang Y. Jiang Y. Zhang M. Xu Z. Wang X. (2023). Mass spectrometry analysis for clinical applications: a review. Crit. Rev. Anal. Chem. 55, 213–232. 10.1080/10408347.2023.2274039 37910438

[B67] Zhang J. Simpson P. C. Jensen B. C. (2021). Cardiac α1A-adrenergic receptors: emerging protective roles in cardiovascular diseases. Am. J. Physiol. Heart Circ. Physiol. 320 (2), H725–H733. 10.1152/ajpheart.00621.2020 33275531 PMC8082792

[B68] Zhao W. Zhao J. Rong J. (2020). Pharmacological modulation of cardiac remodeling after myocardial infarction. Oxid. Med. Cell Longev. 30, 8815349. 10.1155/2020/8815349 PMC7790555 33488934

